# Molecular conservation of metazoan gut formation: evidence from expression of endomesoderm genes in *Capitella teleta* (Annelida)

**DOI:** 10.1186/2041-9139-5-39

**Published:** 2014-10-29

**Authors:** Michael J Boyle, Emi Yamaguchi, Elaine C Seaver

**Affiliations:** Naos Island Laboratory, Smithsonian Tropical Research Institute, Apartado, 0843-03089 Panamá, República de Panamá; Kewalo Marine Laboratory, PBRC/University of Hawaii, 41 Ahui Street, Honolulu, HI 96813 USA; Whitney Laboratory for Marine Bioscience, University of Florida, 9505 Ocean Shore Blvd. St., Augustine, FL 32080 USA

**Keywords:** endoderm, digestive system, gut development, kernel, hybridization, spiralian

## Abstract

**Background:**

Metazoan digestive systems develop from derivatives of ectoderm, endoderm and mesoderm, and vary in the relative contribution of each germ layer across taxa and between gut regions. In a small number of well-studied model systems, gene regulatory networks specify endoderm and mesoderm of the gut within a bipotential germ layer precursor, the endomesoderm. Few studies have examined expression of endomesoderm genes outside of those models, and thus, it is unknown whether molecular specification of gut formation is broadly conserved. In this study, we utilize a sequenced genome and comprehensive fate map to correlate the expression patterns of six transcription factors with embryonic germ layers and gut subregions during early development in *Capitella teleta*.

**Results:**

The genome of *C. teleta* contains the five core genes of the sea urchin endomesoderm specification network. Here, we extend a previous study and characterize expression patterns of three network orthologs and three additional genes by *in situ* hybridization during cleavage and gastrulation stages and during formation of distinct gut subregions. In cleavage stage embryos, *Ct-otx, Ct-blimp1, Ct-bra* and *Ct-nkx2.1a* are expressed in all four macromeres, the endoderm precursors. *Ct-otx, Ct-blimp1,* and *Ct-nkx2.1a* are also expressed in presumptive endoderm of gastrulae and later during midgut development. Additional gut-specific expression patterns include *Ct-otx, Ct-bra*, *Ct-foxAB* and *Ct-gsc* in oral ectoderm; *Ct-otx, Ct-blimp1, Ct-bra* and *Ct-nkx2.1a* in the foregut; and both *Ct-* bra and *Ct-nkx2.1a* in the hindgut.

**Conclusions:**

Identification of core sea urchin endomesoderm genes in *C. teleta* indicates they are present in all three bilaterian superclades. Expression of *Ct-otx, Ct-blimp1* and *Ct-bra*, combined with previously published *Ct-foxA* and *Ct-gataB1* patterns, provide the most comprehensive comparison of these five orthologs from a single species within Spiralia. Each ortholog is likely involved in endoderm specification and midgut development, and several may be essential for establishment of the oral ectoderm, foregut and hindgut, including specification of ectodermal and mesodermal contributions. When the five core genes are compared across the Metazoa, their conserved expression patterns suggest that ‘gut gene’ networks evolved to specify distinct digestive system subregions, regardless of species-specific differences in gut architecture or germ layer contributions within each subregion.

**Electronic supplementary material:**

The online version of this article (doi:10.1186/2041-9139-5-39) contains supplementary material, which is available to authorized users.

## Background

In both protostome and deuterostome clades, sources of endoderm (stomach, intestine, glands) and mesoderm (connective tissue, coelom, somatic gonad, nephridia and most muscle) are commonly derived from a bipotential precursor cell or population of cells, called endomesoderm[1–5]. Networks of transcription factors and cell-signaling molecules have been shown to specify territories of endomesoderm during embryonic development in several model organisms[4, 6–11]. Gene networks that specify endomesoderm are thought to have been in place very early in metazoan evolution[10, 12–14] and appear to contain some network interactions that are highly conserved, as well as some that are evolutionarily labile[15, 16]. Across the Metazoa, orthologs of regulatory genes that specify endomesoderm have different developmental roles during axial patterning and gastrulation[5], cell signaling[17, 18] and germ layer specification[7, 19, 20]. Therefore, variation in the deployment of ancient metazoan network genes may have an important influence on patterning different cell types, organ systems, and ultimately, the morphological diversity of animals[15, 16, 21–24]. Despite the evolutionary implications of this, relatively few studies have examined the expression of endomesodermal network genes in taxa that are distantly related to the standard developmental model systems[4, 12, 16].

*Capitella teleta*, formerly known as *Capitella* sp. I[25] is a marine, polychaete annelid worm and one of several protostome spiralian taxa that develop through a highly conserved, stereotypic program of spiral cleavage[2, 26, 27]. *C. teleta* is proving to be a valuable research organism for investigating fundamental properties of cellular and morphological development[28–32] and patterns of gene expression from embryogenesis through organ system formation in metatrochophore larvae and juvenile worms[33–40]. Additionally, a sequenced genome[41] and comprehensive embryonic fate map[27, 32] have become useful resources for identifying candidate genes and accurately interpreting their expression patterns. Among the spiralian taxa, derivatives of endomesoderm contribute to both endoderm and mesoderm associated with digestive organ systems[2, 26, 42, 43]; however, studies on this diverse group of animals are underrepresented in the context of how or when endomesoderm is genetically specified.

The genetic specification of endoderm and mesoderm in sea urchins[8, 24, 44] represents arguably the most comprehensively described metazoan gene regulatory network (GRN). Within that network, there is a hierarchy of multigene subcircuits that interact to regulate distinct processes during embryogenesis[10]. Upstream of all other subcircuits there is a putative ‘kernel’ of the network that is considered to be the ‘most impervious to change’, unlike more flexible subcircuits within the same GRN[10, 16]. In both sea urchins and sea stars, the endomesoderm ‘kernel’ contains an identical set of core transcription factor genes that regulate the specification of non-skeletogenic mesoderm and most of the gut endoderm within the archenteron during embryonic and larval development[15]. These core transcription factors include Otx, Blimp1/Krox, Brachyury, Foxa, and Gatae[8, 10, 16, 44].

Although the architecture of a GRN cannot be deduced directly from any temporal or spatial patterns of gene expression[44], some patterns should provide a reasonable entry point for detecting evidence that a putative network may be in place. In *C. teleta*, we have previously characterized the expression patterns for orthologs of two core transcription factors, *foxA* and *GATA*, which include three genes in the GATA456 subclass[37]. Those patterns were shown to be consistent with possible roles in specifying embryonic domains of oral ectoderm, endoderm and mesoderm during the process of gut formation. In this study, we investigate the remaining three core transcription factor genes, *orthodenticle* (*Otx*), *Blimp1* and *brachyury* (*Bra*), along with *Nkx2.1*, *goosecoid* (*Gsc*), and *FoxAB* as additional candidates involved in gut formation*.* Orthologs of an *Nkx2.1* gene are expressed in the foregut, midgut and hindgut regions of chordates[45–47], the foreguts of a sea urchin, fly, nematode and a mollusk[48–50], and the posterior ectoderm of an acoel[51]. A *goosecoid* gene is expressed in mesendoderm of a cephalochordate[52], the foreguts of a sea urchin, fly, priapulid, mollusk and polychaete[53–57], and oral ectoderm of an acoel[51]. And *FoxAB*, although found in several invertebrate genomes[58–60], yet without gut-related expression, is consistently assigned to a clade containing FoxA factors, which are known to regulate gut formation across the Metazoa. For all six genes, we present orthology analyses and characterize their expression patterns during development in embryos and larvae of *C. teleta*. With the exception of *Nkx2.1*, there is only one member of each of the transcription factor types in the genome of *C. teleta*, and each one has orthologous gene class members in other metazoan taxa. All six genes have expression patterns associated with development of the digestive organ system. We discuss the identity, expression patterns and potential for interaction of each gene in the context of organ-system development and as components of a putative gene regulatory network in *C. teleta* and other animals.

## Methods

### Animals

A colony of *C. teleta* was maintained in the laboratory according to culturing methods originally established by Grassle and Grassle[61]. Revised protocols for seawater and sediment exchange, temperature-controlled culture conditions, feeding, and the handling of adult worms were conducted according to Seaver *et al*.[33]. Brood tubes from reproductive adult worms were dissected to obtain embryonic and larval stages for the preparation of nucleic acid templates and gene expression protocols. Development from a fertilized egg to a feeding juvenile worm takes approximately 9 days at 18 to 19°C[29, 33].

### Gene isolation and cloning

Total RNA was purified from a pooled sample of early cleavage, blastula, gastrula and larval stages of *C. teleta* with TRI REAGANT™ (Molecular Research Center Inc., Cincinnati, OH, USA). The 5′ and 3′ RACE-Ready cDNA templates (Clontech Laboratories, Mountain View, CA, USA) were constructed from total RNA and then utilized for the rapid amplification of cDNA ends (RACE) with the SMART RACE amplification kit (Clontech Laboratories, Mountain View, CA, USA). Gene-specific RACE primers for *Ct-blimp1*, *Ct-nkx2.1a*, *Ct-foxAB* and *Ct-gsc* were designed from predicted gene models available within the *C. teleta* genome database (http://genome.jgi.doe.gov) at the Joint Genome Institute (JGI)[41]. Gene-specific RACE primers for *Ct-bra* and *Ct-otx* were designed from DNA sequences of degenerate PCR gene products. RACE PCR fragments of each gene were purified from agarose gels, subcloned into pGEM-T Easy vectors (Promega, Madison, WI, USA), and sequenced by Macrogen Inc. (Seoul, South Korea). RACE fragments of the following lengths were isolated and verified: 1616 bp (5′) and 1800 bp (3′) for *Ct-blimp1*; 1534 bp (3′) for *Ct-nkx2.1a*; 1175 bp (5′) and 1347 bp (3′) for *Ct-foxAB*; 893 bp (5′) for *Ct-gsc*; 713 bp (5′) and 1051 bp (3′) for *Ct-bra*; and 1030 bp (3′) for *Ct-otx*.

### Gene sequence alignments and orthology analyses

Homologs of *orthodenticle* (*Otx*), *Blimp1*, *brachyury* (*Bra*) and *goosecoid* (*Gsc*) within the genome of *C. teleta* were identified using the tblastn alignment program, which recovered a single ortholog for each gene. Homologs of Nk2 class genes within the genome of *C. teleta* were identified using blastx, which recovered two *Nkx2.1* orthologs. Amino acid sequence data of orthologs from a diversity of animal taxa were obtained from the protein database within GenBank[62] at NCBI (http://www.ncbi.nlm.nih.gov). Additional amino acid sequence data from the mollusk *Lottia gigantea*, amphioxus *Branchiostoma floridae*, and cnidarian *Nematostella vectensis* were obtained by searching genome databases[41]. Conserved amino acid domains for each gene class were aligned using ClustalX with default parameters in MacVector v11.0 (MacVector, Inc., Cary, NC). Conserved domains included homeodomains of Gsc and Otx, the homeodomain and Nk2-specific domain of NKx2*.* 1a, the zinc finger (C2H2) domain of Blimp1, and the T-box domain of Bra. All of the alignments were edited by hand to correct for errors and analyzed with ProtTest v2.4[63] to determine the appropriate model of protein evolution. The Dayhoff model was recommended for Blimp1, the Jones model for Bra and Otx, and the RtRev model for Gsc and NKx2.1a.

Bayesian and maximum likelihood analyses were performed on all of the amino acid alignments to infer gene orthology assignments for each of the respective candidate gene families. For each of the amino acid alignments, Bayesian analyses were performed with MrBayes v3.1.2[64] using four independent runs, with four chains sampled every 100th generation for 1,000,000 generations; a total of 3,000,000 generations were analyzed for Ct-NKx2.1a. Once convergence was reached, majority rule consensus trees were generated with burnin values of 275 (Blimp1), 225 (Bra), 250 (Gsc), 200 (Otx), or 9,200 (NKx2.1a). Maximum likelihood analyses were performed with RAxML v7.0.0[65] using 1,000 bootstrap replicates and the same gene-specific ProtTest models as for the Bayesian analyses. Nexus alignments are available upon request. Gene trees were visualized with FigTree v1.3.1 (http://tree.bio.ed.ac.uk/software/figtree/) and edited with Adobe Illustrator CS4 (Adobe Systems Incorporated, San Jose, CA, USA). A Bayesian analysis of *Ct-foxAB* orthology was performed previously[66]. Accession numbers for amino acid sequence data in orthology analyses are available as Additional file1: Document S1.

### Whole-mount *in situ* hybridization

Embryonic and larval stages of *C. teleta* were pretreated, fixed and dehydrated according to the methods described by Boyle and Seaver[37]. Whole-mount *in situ* hybridization experiments were performed at 65°C for a period of 72 hours and followed a published protocol[33]. Single-stranded antisense ribonucleic acid probes (riboprobes) were synthesized with the incorporation of digoxigenin-11-uridine-5′-triphosphate (dig-11-UTP; Roche Diagnostics Corporation, Indianapolis, IN, USA) using either a T7 or SP6 MEGAscript kit (Ambion Inc., Austin, TX, USA). Hybridization experiments were performed and replicated with the following gene-specific riboprobe sizes and working concentrations: *Ct-otx*, 1,030 bp at 2.0 ng/μl (embryos), 1.0 ng/μl (larvae); *Ct-blimp1*, 1,616 bp at 1.0 ng/μl (all stages); *Ct-bra*, 1,476 bp at 2.0 ng/μl (embryos), 0.5 ng/μl (larvae); *Ct-nkx2.1a*, 1,534 bp at 2.0 ng/μl (embryos), 1.0 ng/μl (larvae); *Ct-foxAB*, 1,175 bp at 2.0 ng/μl (embryos), 1.0 ng/μl (larvae); and *Ct-gsc*, 893 bp at 2.0 ng/μl (all stages). Riboprobes were detected by chromogenic staining of specimens treated with an anti-digoxigenin-alkaline phosphatase (AP) conjugate antibody and exposed to an enzyme color reaction solution of (4.4 μl of 75 mg/ml nitroblue tetrazolium (NBT):3.3 μl of 50 mg/ml 5-bromo-4-chloro-3indolyl phosphate (BCIP)) per milliliter of AP buffer. Color reactions were terminated with multiple exchanges of PTw (PBS with 0.1% Tween-20 detergent) and processed through a graded series of hybridization buffer/PTw washes to denature residual antibody activity and remove extraneous background. Processed whole-mount *in situ* specimens were stored in glycerol (80% glycerol, 10% 1X PBS, 10% diH_2_0). Replicates of each expression pattern were mounted in glycerol on coated microscope slides (Rain-X™, Sopus Products, Huston, TX, USA) and analyzed under DIC optics with a compound light microscope (Axioskop 2; Carl Zeiss, Inc., Thornwood, NY, USA). Images of selected specimens were captured with a SPOT Flex digital camera (Diagnostic Instruments Inc., Sterling Heights, MI, USA) and edited with Photoshop CS3 (Adobe Systems Incorporated, San Jose, CA, USA). Image stacking of multiple focal planes was performed with Helicon Focus software (Helicon Soft Ltd., Kharkov, Ukraine).

## Results

### Gene orthology analyses

Six candidate transcription factor genes were identified and isolated to examine their patterns of expression and possible roles during development in the marine polychaete annelid, *Capitella teleta* Blake, Grassle & Eckelbarger, 2009. Single copy gene orthologs of *orthodenticle* (*Otx*), *Blimp1*, *brachyury* (*Bra*), *FoxAB* and *goosecoid* (*Gsc*), and two orthologous genes of *Nkx2.1*, were identified in the genome of *C. teleta*[41] (http://genome.jgi.doe.gov). Bayesian (PP, posterior probability) and maximum likelihood (BS, bootstrap) analyses support specific assignments for each of the genes to a distinct clade of orthologs from deuterostomes, ecdysozoans and spiralians. *Ct-otx* was assigned to a clade of PRD class Otx transcription factors [see Additional file2: Figure S1] (PP = 1.0; BS = 99%). *Ct-blimp1* was assigned to a clade of PRDM1/Blimp1 factors [see Additional file3: Figure S2] (PP = 1.0; BS = 85%). *Ct-bra* was assigned to a clade containing other Brachyury/T factors [see Additional file4: Figure S3] (PP = 1.0; BS = 99%). The orthology of *Ct-foxAB* was previously analyzed[66]. *Ct-nkx2.1a* forms a group with *Ct-nkx2.1b* in the Nkx2.1 clade, distinct from a clade of Nkx2.2 homeodomain factors [Additional file5: Figure S4] (pp = 0.96). *Ct-gsc* was assigned to a clade of PRD class Gsc transcription factors [see Additional file6: Figure S5] (PP = 1.0; BS = 64%).

### Brief summary of gut development in *Capitella teleta*

**Figure 1:**
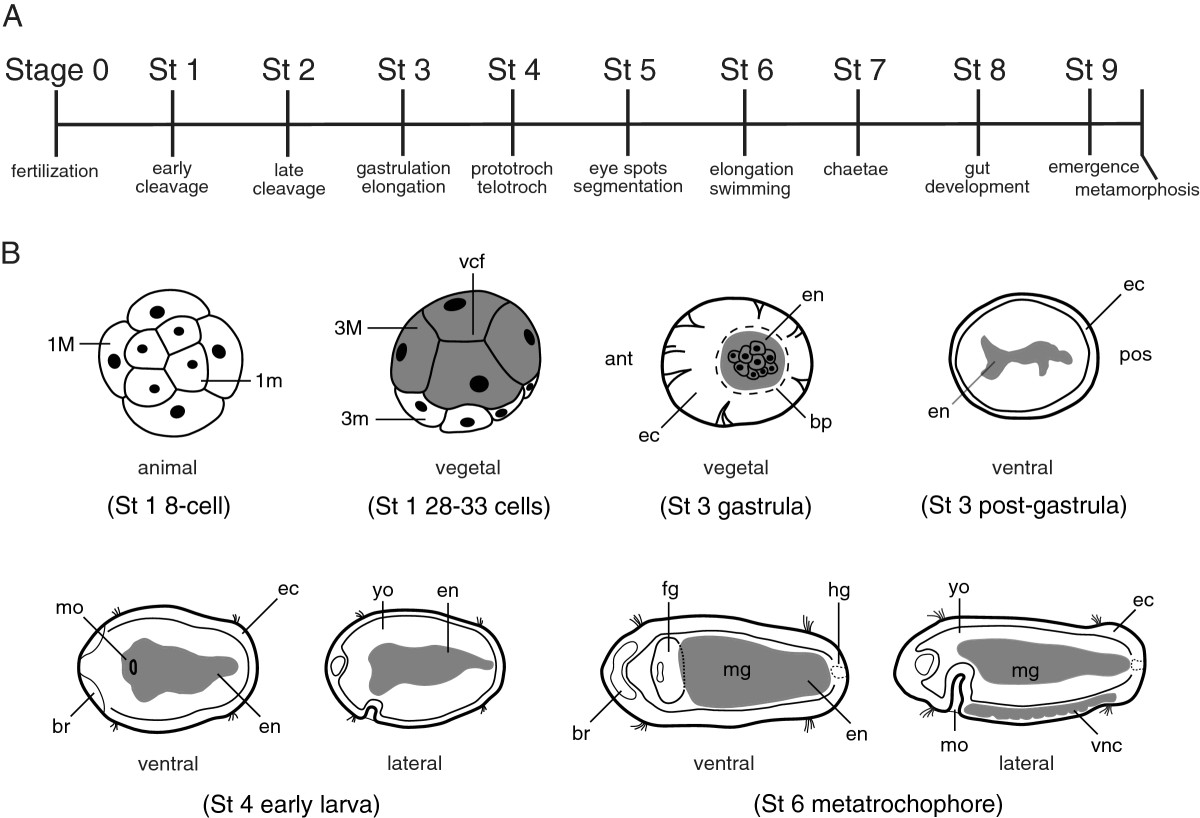
**Schematics of embryonic and larval development in** ***Capitella teleta*** **. (A)** Ten stages of development representing a linear series of events from fertilization through emergence of the stage 9 metatrochophore larva prior to settlement and metamorphosis to a juvenile worm. Stages 1 to 9 each represent approximately 24 hrs at 18 to 19°C. **(B)** Schematics of selected embryonic and larval stages depicting endoderm development and gut formation. Stage 3 schematics are shown with anterior to the left. Stage 4 and stage 6 larvae are oriented in pairs of ventral and lateral views with anterior to the left. Gray shading represents endodermal precursor cells prior to gastrulation and during gastrulation and as definitive endoderm after gastrulation during larval development. View orientations are shown below each embryo and larva. Abbreviations: ant, anterior; bp, blastopore; br, brain; ec, ectoderm; en, endoderm; fg, foregut; hg, hindgut; mo, mouth; pos, posterior; St, stage; vcf, vegetal cross-furrow; vnc, ventral nerve cord; yo, yolk; 1 m, 1st quartet micromere; 1 M, 1st quartet macromere; 3 m, 3rd quartet micromeres; 3 M, 3rd quartet macromeres. Dotted line in stage 3 gastrula indicates the position of the blastopore. Thin solid lines internal to the contour of the body in stages 3, 4 and 6 indicate the inner boundary of regions of ectoderm distinguished by low yolk content.

In *C. teleta*, the developmental life history pattern is indirect and lecithotrophic, whereby adult female worms brood relatively large yolky eggs that develop through a series of embryonic and larval stages (Figure 1). Early embryogenesis exhibits a conserved program of unequal, quartet spiral cleavage[27, 32]. The cleavage program is associated with a stereotypic fate map, and the developmental origins of most tissues are known[27, 32]. The endoderm is derived from four macromeres (3A, 3B, 3C, and 4D) on the vegetal side of the 28- to 33-cell embryo. The macromeres become internalized during gastrulation by epiboly, and their descendants will form the midgut of the alimentary canal. The blastopore completely closes at the end of gastrulation, and a stomodeum forms soon after to establish the definitive mouth. Ectodermal precursors from the 2nd and 3rd quartet micromeres contribute to formation of the mouth, foregut and hindgut, with 2a and 2c descendants contributing to the rectum, 3c and 3d contributing to hindgut musculature, and 4d descendants that form the anus[27]. During foregut development, extensive morphogenesis occurs in the muscular pharynx and the esophagus, which connects the pharynx to the intestine[31, 37]. From anterior to posterior, the gut tube is highly regionalized and is composed of a mouth, foregut (buccal cavity, pharynx, and esophagus), midgut (intestine), hindgut (rectum) and anus[27, 31, 37]. Organogenesis of the gut occurs over a period of several days during larval development, and the process is not complete until nonfeeding metatrochophore larvae emerge from the brood tube at stage 9[27, 30, 37]. Feeding commences within 24 hours following settlement and metamorphosis to a juvenile worm.

## Whole-mount *in situ* expression patterns

### Orthodenticle

**Figure 2:**
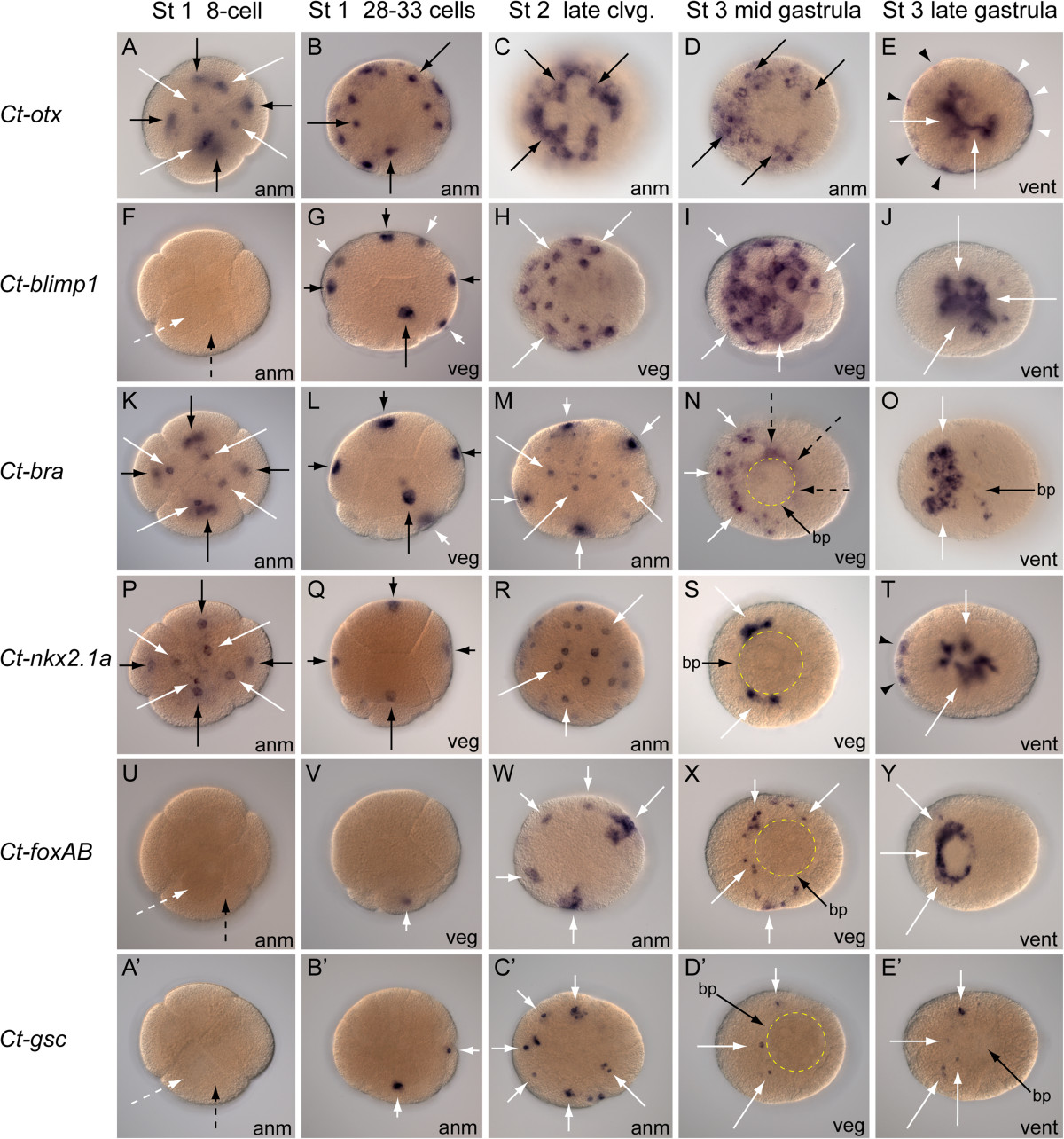
**Expression of** ***Otx, Blimp1, Bra, Nkx2.1a, FoxAB*** **and** ***Gsc*** **during embryogenesis in** ***Capitella teleta*** **.** Each row shows expression (blue color) for the single gene listed at the left margin. The approximate stage of development for the embryos in each column is listed at the top margin. The orientation of each embryo is listed at the bottom right corner of every panel (anm, animal view; veg, vegetal view; vent, ventral view). In embryos with approximately 28 to 33 cells, the D quadrant is toward the bottom of each panel. Yellow dotted lines indicate the position of the blastopore. M is the animal view of L; R is the animal view of Q. Anterior is to the left in all Stage 3 embryos. **(A-E)** *Ct-otx* is expressed in each macromere (black arrows) and micromere (white arrows) of 8-cell embryos **(A)**, in micromeres (black arrows) encircling the animal pole **(B-D)**, in both anterior (black arrowheads) and posterior (white arrowheads) ectoderm and within and around the blastopore (white arrows) of gastrulae **(E)**. **(F-J)** *Ct-blimp1* is not detected in macromeres (dashed black arrow) or micromeres (dashed white arrow) of 8-cell embryos **(F)**; *Ct-blimp1* is expressed in macromeres (black arrow) and 4q micromeres (white arrow) of 28- to 33-cell embryos **(G)**, in vegetal cells (white arrows) of stage 2 and stage 3 embryos **(H-I)**, and in the endoderm **(J)**. **(K-O)** *Ct-bra* is expressed in each macromere (black arrows) and micromere (white arrows) of 8-cell embryos **(K)**, in macromeres (black arrows) and micromeres (white arrows) in each quadrant of 28- to 33-cell embryos **(L-M)**, anterior to the blastopore (short white arrows) and around the blastopore (dashed black arrows) of gastrulae **(N)**, and on the ventral anterior surface of late gastrulae **(O)**. **(P-T)** *Ct-nkx2.1a* is expressed in each macromere (black arrows) and micromere (white arrows) of 8-cell embryos **(P)**, in macromeres (black arrows) and micromeres (white arrows) of 28- to 33-cell embryos **(Q-R)**, along the left and right sides of the blastopore **(S)**, and in both anterior ectoderm (black arrowheads) and endoderm (white arrows) of late gastrulae **(T)**. **(U-Y)** *Ct-foxAB* is not detected in macromeres (dashed black arrow) or micromeres (dashed white arrow) of 8-cell embryos **(U)**; *Ct-foxAB* is expressed in a single D-quadrant cell (white arrow) at the 28-cell stage **(V)**, in each quadrant (white arrows) on the animal hemisphere of stage 2 **(W)**, outside the blastopore (white arrows) in vegetal micromeres **(X)**, and in anterior surface cells (white arrows) encircling the site of stomodeum formation **(Y)**. **(A’-E’)** *Ct-gsc* is not detected in macromeres (dashed black arrow) or micromeres (dashed white arrow) of 8-cell embryos **(A’)**; *Ct-gsc* is expressed in two animal micromeres (white arrows) within embryos of approximately 28 cells **(B’)**, in each quadrant (white arrows) on the animal hemisphere of stage 2 **(C’)**, in several micromeres (white arrows) anterior to the blastopore **(D’)** and in micromeres (white arrows) on the anterior surface of late gastrulae **(E’)**. bp, blastopore; St, stage. The image in each panel was created by combining micrographs from a series of focal planes.

**Figure 3:**
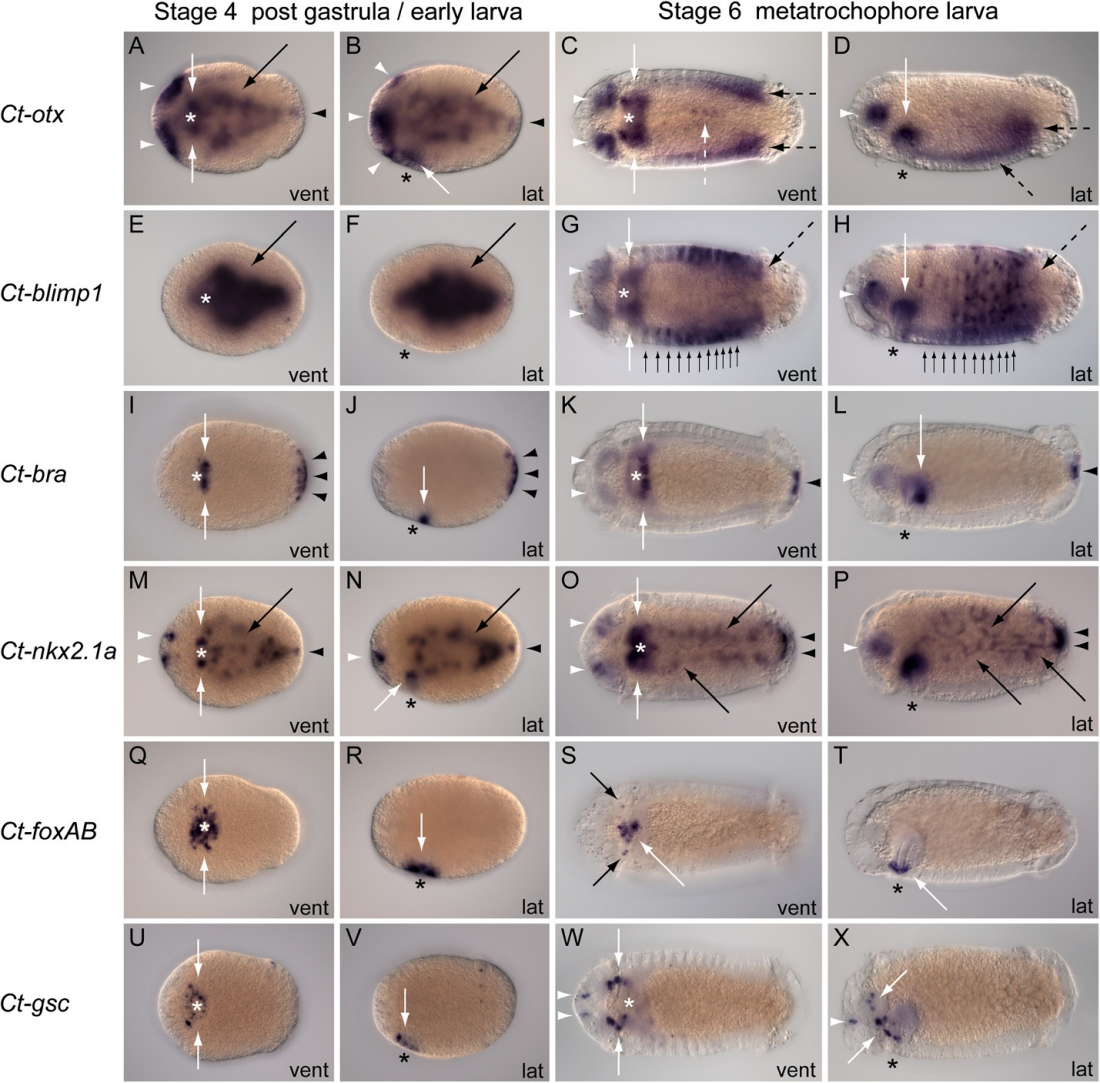
**Expression of** ***Otx, Blimp1, Bra, Nkx2.1a, FoxAB*** **and** ***Gsc*** **during larval development in** ***Capitella teleta*** **.** Each row shows expression (blue color) for the single gene listed at the left margin. The stage of development for the larvae in each column is listed at the top margin. The orientation of each larva is listed at the bottom right corner of every panel (vent, ventral view; lat, lateral view). For each row, columns 1 and 2 are different views of the same stage 4 larva; columns 3 and 4 are different views of the same stage 6 larva. Anterior is to the left in all panels; ventral is down for each lateral view. A black or white asterisk marks the position of the mouth. **(A-B)** *Ct-otx* is expressed in anterior ectoderm, including the brain (white arrowheads), in the stomodeum (white arrows) endoderm (black arrows) and hindgut primordia (black arrowheads) at stage 4. **(C-D)** *Ct-otx* is expressed in the brain (white arrowheads) foregut (white arrows) posterior segments (dashed black arrows) and surface cells on the ventral midline (dashed white arrow) at stage 6. **(E-H)** *Ct-blimp1* is expressed in endoderm (black arrow) at stage 4, and in the brain (white arrowheads) foregut (white arrows) most segments of the trunk (black arrows) and the posterior growth zone (dashed black arrows) at stage 6. **(I-L)** *Ct-bra* is expressed on the posterior side of the foregut (white arrows) and in ectoderm at the posterior-most end of the body (black arrowheads) of stage 4 larvae, and in the brain (white arrowheads) foregut (white arrows) and anus (black arrowheads) at stage 6. **(M-P)** *Ct-nkx2.1a* is expressed in the brain (white arrowheads) foregut (white arrows) endoderm (black arrow) and hindgut (black arrowheads) in stage 4 and stage 6 larvae. **(Q-T)** *Ct-foxAB* is expressed primarily in surface cells around the mouth (white arrows) and also lateral to the mouth (black arrows, S) at stage 4 and stage 6. **(U-V)** *Ct-gsc* is expressed in surface cells around the mouth (white arrows) at stage 4. **(W-X)** *Ct-gsc* is expressed in ectoderm cells anterior to the brain (white arrowheads) and in several anterior cells flanking each side of the brain and foregut (white arrows) in stage 6 larvae. The image in each panel was created by combining micrographs from a series of focal planes.

The expression of *orthodenticle* (*Ct-otx*) is initially detected in 8-cell embryos. Expression is relatively higher in the C and D macromeres; however, *Ct-otx* appears to be expressed in all four macromeres and first quartet micromeres (Figure 2A). The expression of *Ct-otx* was observed to be associated with both daughter cells of the first quartet micromeres (1q) during division. After the 1q division, *Ct-otx* is detected in the 1q^2^ daughter micromeres but not the animal daughter cells (1q^1^) of 16- to 20-cell embryos (not shown). In embryos containing 28 to 33 cells (stage 1), *Ct-otx* is expressed in 3A to 3C macromeres and 2nd quartet micromeres, with low levels of expression in the 4D macromere and some 2d daughter micromeres. Notably, no expression is detected in 1q micromeres at this stage (Figure 2B). In stage 2 embryos containing 30 to 40 cells, there is a distinct ‘cross-shaped’ expression pattern on the animal pole where *Ct-otx* is expressed in 2nd and 3rd quartet derivatives, but not detected in any 1st quartet micromere lineages (Figure 2C). There is expression in macromeres at this stage, although expression of *Ct-otx* in the D quadrant is low or undetectable when compared with blastomeres from all other quadrants. During mid-gastrulation (Figure 2D), there is a circular pattern of *Ct-otx* expression on the animal hemisphere with a gap of expression within the D quadrant and expression both on and within the blastopore and in ectoderm at either end of the embryo [see Additional file7: Figure S6 A, B]. In late gastrulae, ectodermal expression domains are detectable at both anterior and posterior ends of the embryo and within both surface and subsurface cells at the region of the blastopore (Figure 2E). In stage 4 larvae, *Ct-otx* is expressed in a bilateral pattern in the anterior ectoderm associated with brain anlagen, in domains of head ectoderm outside the brain, and on left, right and posterior sides of the stomodeum (Figure 3A, B). *Ct-otx* is also expressed internally within endoderm cells extending from the position of the stomodeum to the hindgut at the posterior end of the larva. There is a gap in *Ct-otx* expression internally between the stomodeum and midgut endoderm (Figure 3B). In stages 6 and 7, *Ct-otx* is expressed in bilateral lobes of the brain, the developing foregut, lateral-posterior ectoderm within the posterior growth zone of the segmented trunk, and within a few cells of the ventral nerve cord along the midline (Figure 3C, D). At stage 9, *Ct-otx* is expressed in the brain, foregut, ventral nerve cord and posterior growth zone (not shown).

### B lymphocyte-induced maturation protein 1

The earliest expression of *B lymphocyte-induced maturation protein 1* (*Ct-blimp1*) is detectable in seven blastomeres of 28- to 33-cell embryos. In these stage 1 embryos, *Ct-blimp1* is expressed in 4A to 4D macromeres and 4a to 4c micromeres (Figure 2G). During stage 2, most of the blastomeres on the vegetal hemisphere show *Ct-blimp1* expression, with the exception of the D-quadrant (Figure 2H). There are two or three blastomeres of the D-quadrant on the animal hemisphere that show expression, although *Ct-blimp1* is not detectable in any other cells on the animal hemisphere at this stage. Embryos undergoing epiboly during gastrulation show positive *Ct-blimp1* expression in approximately 18 to 20 cells on the vegetal plate, including the conspicuously large macromeres (Figure 2I). During gastrulation, transcripts are detectable in both nuclear and cytoplasmic regions of vegetal cells, which become localized around and within the blastopore between the mid- and late gastrulation stages [see Additional file7: Figure S6 C, D]. In late gastrulae of stage 3, *Ct-blimp1* expression is concentrated on the ventral side in the endoderm (Figure 2J). In this region, *Ct-blimp1* is expressed in surface and subsurface cells associated with the blastopore and in one or two surface cells that are posterior to the blastopore (Figure 2J). In stage 4 larvae, *Ct-blimp1* is expressed internally within the endoderm along the anterior-posterior (A/P) axis, and there is no detectable expression in any surface cells (Figure 3E, F). The same pattern is observed in stage 5 larvae, with the addition of low-level expression in the brain and foregut [see Additional file7: Figure S6 F, G]. At stage 6, the expression of *Ct-blimp1* in midgut endoderm is diminished, and there are new expression domains in the bilateral lobes of the brain, throughout most of the developing foregut, in a segmental pattern along the trunk extending from the foregut to the telotroch, and from the ventral side to the dorsal side (Figure 3G, H). At stage 8, *Ct-blimp1* is expressed in the brain, foregut and posterior growth zone (not shown).

### Brachyury

The expression of *brachyury* (*Ct-bra*) is initially detected in all cells of the 8-cell embryo (Figure 2K). In stage 1 embryos with 28 to 33 cells, *Ct-bra* is expressed in A-D macromeres and in a subset of second and third quartet micromeres from each quadrant (Figure 2L, M). In stage 1 embryos for which the color reaction was extended over a longer time period, labeling was detected in cells at the animal pole that may indicate expression in first quartet micromeres (not shown). During gastrulation, *Ct-bra* is consistently expressed in surface and subsurface cells on left-lateral and posterior sides of the blastopore in an asymmetric pattern and in surface cells that are spread across the ventral-anterior side of the embryo, distinct from blastoporal expression (Figure 2N). At the completion of gastrulation, *Ct-bra* is expressed in a semicircular pattern of surface cells anterior to the blastopore and in several cells on left and right sides of the midventral surface of the embryo (Figure 2O). In stage 4 larvae, *Ct-bra* is differentially expressed on the posterior side of the stomodeum and in surface cells at the posterior end of the larval body (Figure 3I, J). With longer color development, *Ct-bra* expression is detected in anterior ectoderm, in both surface and subsurface cells along ventrolateral sides of the body and in the endoderm [see Additional file7: Figure S6 E]. During stages 6 to 7, *Ct-bra* is expressed in the brain, in subsurface cells along each side of the midline on the posterior face of the foregut, and in the anus (Figure 3K, L), as well as in mesoderm along the ventrolateral sides of the trunk [see Additional file7: Figure S6 H].

### Nk2.1

The expression of *Ct-nkx2.1a,* one of two *Nk2.1* genes in *C. teleta*, is observed in each macromere and micromere of the 8-cell embryo (Figure 2P). In 28- to 33-cell embryos, *Ct-nkx2.1a* is expressed in most cells except for some 2d lineage micromeres (Figure 2Q, R). During gastrulation, the only expression is in bilateral domains flanking two sides of the blastopore (Figure 2S). In late stage 3 gastrulae, *Ct-nkx2.1a* expression is detected in paired clusters of 1 to 3 labeled cells in the anterior ectoderm and in the endoderm (Figure 2T). Stage 4 larvae show *Ct-nkx2.1a* expression in four bilateral domains. These include cell clusters in anterior ectoderm, a subset of subsurface stomodeal cells, broadly dispersed internal presumptive midgut cells, and a distinct subsurface domain at the posterior end (Figure 3M, N). Similar expression domains persist during stage 5. In stage 6 to 7 larvae, *Ct-nkx2.1a* is expressed in a subset of brain cells, in dorsal-anterior foregut tissue, within ‘web-like’ expression along the length of the midgut, and in the rectum at the posterior terminus of the midgut (Figure 3O, P).

The expression of the second *C. teleta Nk2.1* gene, *Ct-nkx2.1b*, was characterized during larval stages. In early stage 4 larvae, *Ct-nkx2.1b* is detected in the two nascent brain lobes and in subsurface cells of the presumptive foregut [see Additional file8: Figure S7 A, B]. By stage 5, additional expression appears as a broad ventrolateral domain in the trunk ectoderm and mesoderm that extends from posterior of the mouth to the telotroch [see Additional file8: Figure S7 C]. Expression in the brain and foregut persists at this stage. At stage 6, the trunk expression domains have expanded circumferentially and meet at the ventral midline, and the mesoderm expression extends slightly posterior of the ectoderm expression, beneath the telotroch [see dashed arrows in Additional file8: Figure S7 D]. *Ct-nkx2.1b* is also detectable in the hindgut [see the arrowhead in Additional file8: Figure S7 D]. Expression in the brain, foregut and trunk is still present in stage 6 larvae. In summary, each of the two *Nk2.1* genes of *C. teleta* has a unique expression pattern. In the larval trunk, *Ct-nkx2.1a* is expressed in the endoderm whereas *Ct-nkx2.1b* is present in the ectoderm and mesoderm, although both *Ct-nkx2.1a* and *Ct-nkx2.1b* are expressed in the brain, foregut and hindgut.

### Forkhead box A/B

The expression of *forkhead box A/B* (*Ct-foxAB*) is first detectable in 28- to 33-cell embryos. At this stage, *Ct-foxAB* is expressed within the D-quadrant, in two distinct cells of the 2d lineage that are most likely 2d^11^ and 2d^12^ (Figure 2V). In late stage 1 embryos with 50 to 60 cells, *Ct-foxAB* is expressed in one cell, or pairs of cells, in each quadrant on the animal hemisphere. Stage 2 late cleavage embryos show expression in several cells of each quadrant on lateral margins of the animal hemisphere (Figure 2W). During mid-gastrulation, *Ct-foxAB* is expressed in multiple surface cells surrounding anterior and lateral sides of the blastopore on the vegetal hemisphere (Figure 2X). Late gastrulae show *Ct-foxAB* expression in surface cells on anterior and lateral sides surrounding the position where the stomodeum will form (Figure 2Y). With development of stage 4 larvae, *Ct-foxAB* expression is limited to surface cells surrounding the stomodeum, in a band 2 to 3 cells wide (Figure 3Q, R). The expression of *Ct-foxAB* in stage 6 larvae is predominantly in subsurface oral ectoderm surrounding the buccal tube (Figure 3S, T). Additional expression is in a bilateral pair of cell clusters positioned lateral to the mouth and at low levels in internal epithelia of the stomodeum. (Figure 3S, T). In stages 7 to 8, there is a low level of *Ct-foxAB* expression in ectoderm of the brain, foregut, and mouth and in mesoderm of the posterior growth zone within posterior segments of the trunk [see Additional file7: Figure S6 I].

### Goosecoid

The expression of *goosecoid* (*Ct-gsc)* is first detectable in stage 1 embryos with approximately 30 cells. *Ct-gsc* is expressed at this stage on the animal hemisphere in one cell from each of the C and D quadrants (Figure 2B’). In embryos with 47 to 56 cells, expression is detected on the animal hemisphere in a minimum of one cell from each of the C and D quadrants and typically 1 to 2 cells from the A or B quadrant (not shown). In each embryo, the expressing cells appear to be 3rd quartet micromeres. Stage 2 embryos express *Ct-gsc* on the animal hemisphere in several cells from each of the four quadrants (Figure 2C’), and no expression is observed on the vegetal hemisphere. During both mid- and late gastrulation, *Ct-gsc* is expressed in a small number of surface cells across the ventral-anterior face of the embryo, outside of the blastopore (Figure 2D’, E’). In stage 4 larvae, *Ct-gsc* is expressed in a ring of surface cells surrounding the mouth and cells extending laterally from the anterior side of the mouth (Figure 3U, V). The stage 5 expression pattern includes bilateral clusters of 1 to 2 cells each on the surface between the mouth and prototroch and in discrete cells on left and right sides lateral to the brain. In stages 6 to 7, *Ct-gsc* is expressed in multiple cells and cell groups at the position of the circumesophageal connectives, where the expressing cells appear to extend from each side of the brain in a ventral-posterior direction across lateral sides of the buccal cavity toward the ventral nerve cord (Figure 3W, X). There is also a bilateral pair of *Ct-gsc*-positive cells in the anterior ectoderm (Figure 3W, X). Several cells in each of these anterior domains have a distinctive elongate morphology.

## Discussion

### Gene orthologs of the sea urchin endomesoderm kernel in *Capitella teleta*

We have identified a single ortholog each of *Otx*, *Blimp1* and *Brachyury* transcription factor genes from the genome of *C. teleta*, which supplements previous work characterizing both *foxA* and *GATA* factors from the same species[37]. This demonstrates that all five regulatory genes in the ‘kernel’ of the sea urchin endomesoderm GRN are present in an annelid, which is consistent with previous records for subsets of these orthologs within Spiralia[50, 55–57, 67–75] and thus confirms their presence in all three superclades of the Bilateria. These five genes are also in the genome of an anthozoan cnidarian, *Nematostella vectensis*[14, 76], indicating that they were most likely present in a primitive metazoan ancestor prior to the divergence of Cnidaria and Bilateria. Based on the orthology assignments for each of the five individual genes (tree figures in this paper; and[66]), it is clear that these gene families are broadly conserved across the metazoan tree of life[51, 77], although the number of identified genes varies within particular taxonomic groups.

There are three *Otx* orthologs in *N. vectensis* but fewer in other cnidarians[78], two Otx proteins in a sea urchin[79], at least three Otx proteins in a sea star[80] and several copies in vertebrates despite evidence for a single copy of *Otx* in an ancestral chordate[81]. Among spiralians, there is no less variation in the number of orthologs, with one in *P. dumerilii*[55] and two in *Hydroides elegans*[70], and we identified at least three *Otx* paralogs in the genome of a leech, *Helobdella robusta*[41]. This demonstrates that *Otx* genes have experienced duplication, diversification and perhaps loss in both bilaterian and non-bilaterian groups[79, 81]. There are comparatively fewer confirmed records of *Blimp1* orthologs available; however, there is one gene in the genome of *L. gigantea,* two genes in the *H. robusta* genome[41], and one gene in the fruit fly genome (http://flybase.org), and paralogs have been identified in several vertebrates, especially primates[82], indicating they are present in each of the three major bilaterian clades. Outside Bilateria, one *Blimp1* transcript (*Nvblimp-like*) was identified in a cnidarian[14]. Of the many T-Box family genes, single copies of *brachyury* are predominantly found among protostomes, with multiple copies present in several deuterostome clades[83]. Therefore, although the five ‘kernel’ orthologs, including *FoxA* and *GATA*[37, 66, 84, 85], appear to have undergone taxon-specific evolutionary changes in the number of genes or protein products that are present, they have been retained across a broad diversity of animal genomes.

Of the additional three transcription factors isolated from *C. teleta*, orthologs of *NK2* homeobox genes are found in a sponge and two cnidarians[86–89], *goosecoid* is also found in a cnidarian[90], and both genes have been identified in the genomes of many bilaterians (see[51]) including ecdysozoans[48, 49, 53] spiralians[50, 55, 56] and chordates[45, 47, 52]. Interestingly, there are two *Nk2.1* genes (*Ct-nkx2.1a* and *Ct-nkx2.1b*) in the genome of *C. teleta*, which likely result from a clade-specific duplication event. And though only a few records of *FoxAB* factors are published, this gene is found in both protostome and deuterostome clades[58, 60, 91]. All of the genes discussed here, whether they are known members of an established regulatory network[4, 8, 10, 13, 20] or not, are considered to be associated with gut formation.

### Conserved endodermal expression patterns without a definitive ‘endomesoderm’

Endoderm in the sea urchin, *C. elegans* and spiralians is generally derived from a bipotential ‘endomesoderm’. In the sea urchin, embryonic endomesoderm gives rise to gut endoderm and several types of mesoderm[8, 44, 92]. In the embryo of *C. elegans*, endomesoderm generates the intestine and part of the muscular pharynx[4, 93]. Spiralian endomesoderm is an embryonic precursor of some endoderm of the intestine and most of the adult mesoderm[1, 2, 43]. The endoderm derived from endomesoderm territories in the sea urchin is specified at the blastula stage from two sister lineages, veg_2_ and veg_1_ micromeres[8, 11, 94], whereas all of the endoderm in *C. elegans* is derived from the ‘E’ cell, a single daughter of the EMS cell in a 4-cell embryo[4, 93, 95], and in most spiralians, endomesoderm is typically formed from the 4d micromere, the mesentoblast[2]. In *C. teleta*, the 4d micromere is not a true mesentoblast. In fact, there is no single cellular precursor for endomesoderm; all endodermal tissues develop from 3A, 3B, 3C and 4D macromeres, and the mesodermal bands originate from the 3c and 3d micromeres[27]. Yet regardless of whether a true bipotential endomesoderm is broadly conserved across these and other animals, and despite important cellular and developmental differences between a sea urchin, a nematode, a polychaete and other spiralians, the deployment of endomesodermal gene orthologs that likely specify tissues of digestive organ systems is notably similar.

Orthologs of the core set of five transcriptional regulators in the sea urchin endomesoderm GRN are expressed in overlapping domains in *C. teleta* during a similar developmental period (Figure 3A, B). In embryos containing 28 to 32 cells, all five genes (*Ct-otx, Ct-blimp1, Ct-bra, Ct-foxA, Ct-gataB1*) are expressed in 3rd quartet macromeres on the vegetal pole, which is most likely during endoderm specification. Co-expression of these five genes suggests they could be involved in regulatory interactions in each of the 3Q macromeres that contribute to endoderm in *C. teleta*[27]. Each one of the five orthologs is also expressed within and around the blastopore and in cells that invaginate during gastrulation when the endoderm is segregated from the other two germ layers (Figures 1 and3A). During closure of the blastopore and formation of the early larva at stage 4, each ortholog is also detected internally, within endoderm of the presumptive midgut territory[37]. Thus, the co-expression patterns of *Ct-otx, Ct-blimp1, Ct-bra, Ct-foxA* and *Ct-gataB1* are spatially and temporally correlated with endoderm specification, gastrulation and the presumptive midgut, a major component of the digestive organ system in *C. teleta*. These results provide some evidence that endoderm formation in this polychaete species may be under the control of a core gene regulatory network, similar to what is characterized in the sea urchin[8, 44] and a sea star[15] and with some commonalities to what is shown in *C. elegans*[20]. Recent expression profiles and functional experiments in the emerging cnidarian GRN model show that three of the five ‘kernel’ genes (*Nvotx*, *Nvbra*, and *NvfoxA*) may have a crucial role in specifying a bifunctional, endomesoderm-like gastrodermis[14]. This indicates that at least a portion of the kernel may have been in place in the cnidarian-bilaterian ancestor. To move the spiralian polychaete system toward direct comparisons with echinoderms, *C. elegans* and *N. vectensis*, functional perturbations of gene expression along with qPCR will be needed to confirm or refute our inference of a putative endodermal GRN in *C. teleta*. However, the developmental expression patterns of all five ‘kernel’ orthologs that we have characterized, along with previously published expression patterns for additional orthologs of the sea urchin endomesoderm GRN, provide a strong list of candidate genes with potential to function during endoderm specification in *C. teleta*.

### Gene expression and cell fate support multiple roles during gut development in *Capitella teleta*

**Figure 4:**
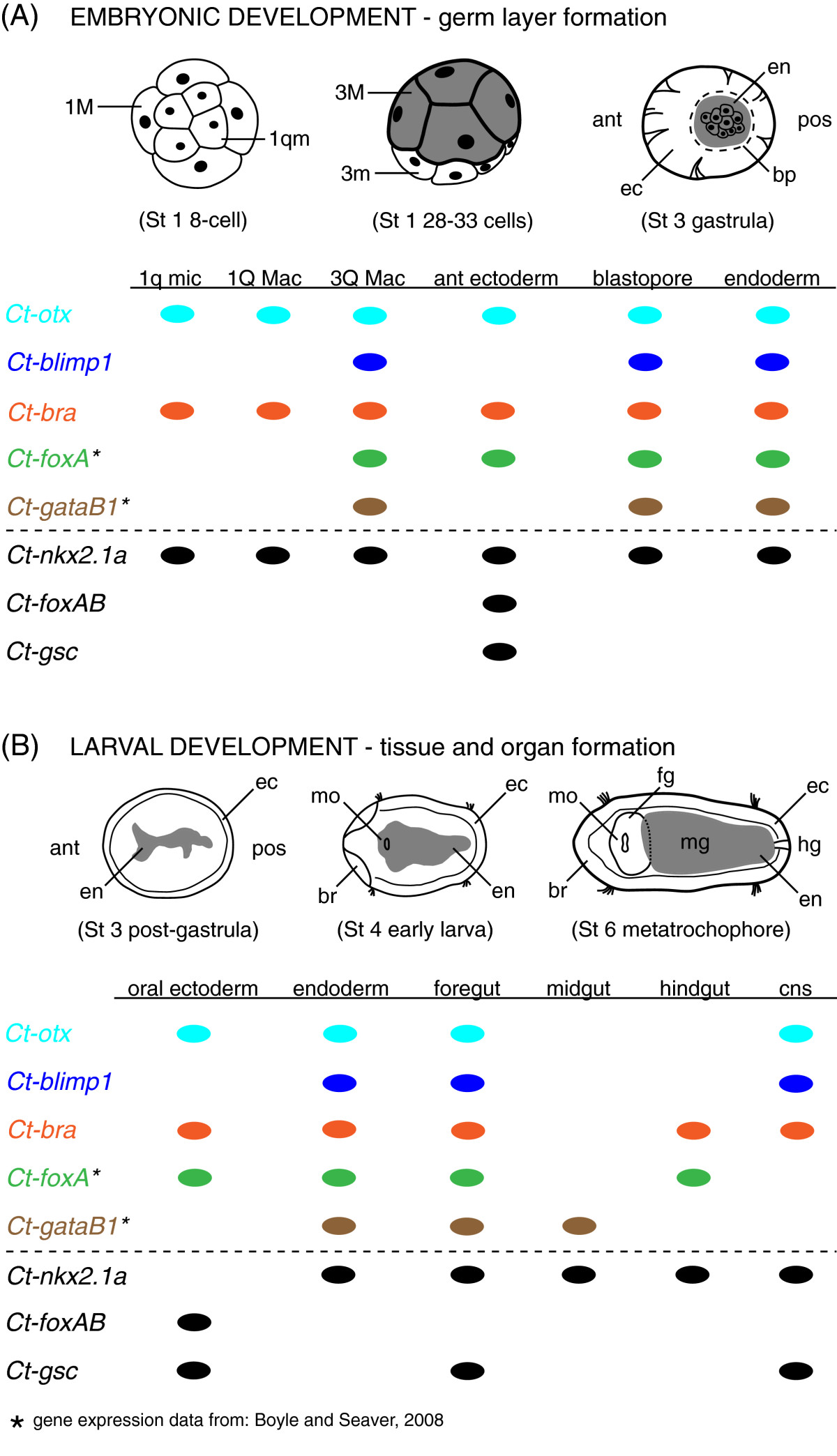
**Comparative summary of gene expression in cell and organ territories during embryonic and larval development in** ***Capitella teleta.*** **(A)** Schematic illustrations of early embryonic stages and a table comparing the presence or absence of detectable gene expression in selected blastomere and germ layer regions. **(B)** Schematic illustrations of post-gastrula and larval stages and a table comparing the presence or absence of gene expression in selected tissues and organs associated with gut formation. Schematic stages are oriented in ventral view with anterior to the left. The colored names and ovals identify *C. teleta* orthologs of the five core genes in the endomesoderm GRN ‘kernel’ of the sea urchin. The five orthologs in **(A)** and **(B)** are separated by a dashed line from three additional *C. teleta* genes characterized in this study. The gray shading represents endoderm. Abbreviations: ant, anterior; bp, blastopore; br, brain; cns, central nervous system; ec, ectoderm; en, endoderm; fg, foregut; hg, hindgut; mg, midgut; mo, mouth; pos, posterior; St, stage; 1 m, 1st quartet micromere; 1 M, 1st quartet macromere; 3 m, 3rd quartet micromeres; 3 M, 3rd quartet macromeres. Dotted line in stage 3 gastrula indicates the position of the blastopore. Thin solid lines internal to the contour of the body in stages 3, 4 and 6 indicate the inner boundary of regions of ectoderm distinguished by low yolk content.

Each of the genes isolated in this study exhibit patterns of expression that are consistent with one or more roles during gut development in *C. teleta*. Both *Ct-foxAB* and *Ct-gsc* show specific expression associated with oral ectoderm, although they are clearly in separate subdomains. *Ct-foxAB* is the only gene of *C. teleta* that appears to be restricted to the presumptive stomodeum during gastrulation and larval development (Figures 2 and3). This would imply its involvement in mouth formation, although expression was not observed in 3a, 3b, or 3c blastomeres, which are known to form the mouth[27]. Hence, other transcription factors upstream of *Ct-foxAB* may influence stomodeal development in *C. teleta. Ct-gsc* appears to be expressed in a subset of anterior neurons associated with the stomodeum and foregut, and may be involved in development and differentiation of the circumesophageal connectives and neuronal subtype identity. This is consistent with *goosecoid* expression in oral ectoderm of an acoel flatworm[51] and cells associated with the stomodeum in *Drosophila*[53] and two spiralians (*P. vulgata*[56] and *P. dumerilii*[55]). When considered together, these data suggest a conserved role for *Gsc* orthologs in the stomotogastric component of the central nervous system in protostomes, which is distinct from its conserved organizer function in vertebrates[96, 97]. Based solely on expression patterns, *Ct-foxAB* and *Ct-gsc* appear to be disconnected from potential network interactions with the other transcription factors that are likely to be involved in endoderm or foregut development. It is interesting that in the sea urchin, *SpGsc* promotes oral ectoderm differentiation within the ectoderm gene network[54] (http://sugp.caltech.edu/endomes/), and therefore, *Ct-foxAB* and *Ct-gsc*, along with *Ct-otx*, *Ct-bra* and *Ct-foxA,* may be transcriptional regulators within an analogous ectoderm network in Spiralia (Figure 4B).

The expression patterns of *Ct-otx*, *Ct-blimp1*, *Ct-bra* and *Ct-nkx2.1a* indicate they each have specific roles in foregut and midgut development and may be part of a specification network in one or both of those regions. *Ct-otx*, *Ct-bra* and *Ct-nkx2.1a* are expressed in 2nd quartet micromeres, which contribute to the foregut[32], and in the stomodeum after gastrulation. During larval development, all four are expressed within foregut tissues, which are formed from ectodermal and mesodermal germ layers, but not endoderm. And along with *Ct-foxA* and *Ct-gataB1*[37], *Ct-otx*, *Ct-blimp1*, *Ct-bra, Ct-nkx2.1a* and *Ct-nkx2.1b* are deployed in overlapping domains during foregut morphogenesis. In addition, it should be noted that many other genes are expressed in the developing foregut of *C. teleta*, including *parahox* genes[34], pair-rule genes[98], and mesoderm patterning genes[35, 39], which reflect the complex developmental control of this organ[31, 37]. What is also significant about the expression patterns in this study is that most of them are detected over a period of several days, starting from early embryogenesis in gut precursors and persisting through gut morphogenesis during larval development (Figure 3). Sustained expression may indicate that particular transcription factors are required to control an entire developmental process, including gastrulation or the organization of groups of cells into specific tissues, although this hypothesis would need verification by functional experiments. Furthermore, they are expressed in different germ layers and organ systems, including the patterns we observed for *Ct-otx, Ct-blimp1, Ct-bra*, *Ct-nkx2.1a* and *Ct-nkx2.1b* in the nervous system. This implies that they are likely to be important for specification events in the foregut, as well as specification and patterning events in other tissues. However, individual genes often exhibit different roles during the course of animal development, and knowledge of the evolutionary history of a gene is necessary to imply gene-specific evidence of gene co-option[99–101].

The early expression of *Ct-otx*, *Ct-bra* and *Ct-nkx2.1a* in 1q micromeres and 1Q macromeres has additional implications for gut-related network interactions (Figures 2 and4A). They are detectable in 8-cell embryos of *C. teleta* just prior to transcription of *Ct-bra*, *Ct-foxA* and *Ct-gataB1* at the blastula stage. It is during a similar stage of development when orthologs of *Otx* provide *cis*-regulatory inputs to *GataE*, *Blimp1*, *Brachyury* and *FoxA* transcriptional regulators of the sea urchin endomesoderm GRN to initiate the specification of endoderm and oral ectoderm of the larval foregut[11, 44, 102]. The transcription factors *Ct-bra*, *Ct-nkx2.1a*, *Ct-nkx2.1b* and *Ct-foxA*[37] also exhibit overlapping expression patterns in the hindgut, which could represent another potential site of gut-specific, gene regulatory network interactions that should be investigated further. When considered together, this particular suite of DNA-binding genes are expressed in *C. teleta* along the alimentary canal in foregut, midgut and hindgut regions during periods of germ layer specification and gut morphogenesis before metatrochophore larvae settle and transform into feeding juvenile worms.

### Evidence of similarity in endomesoderm specification from gene expression in Spiralia

**Figure 5:**
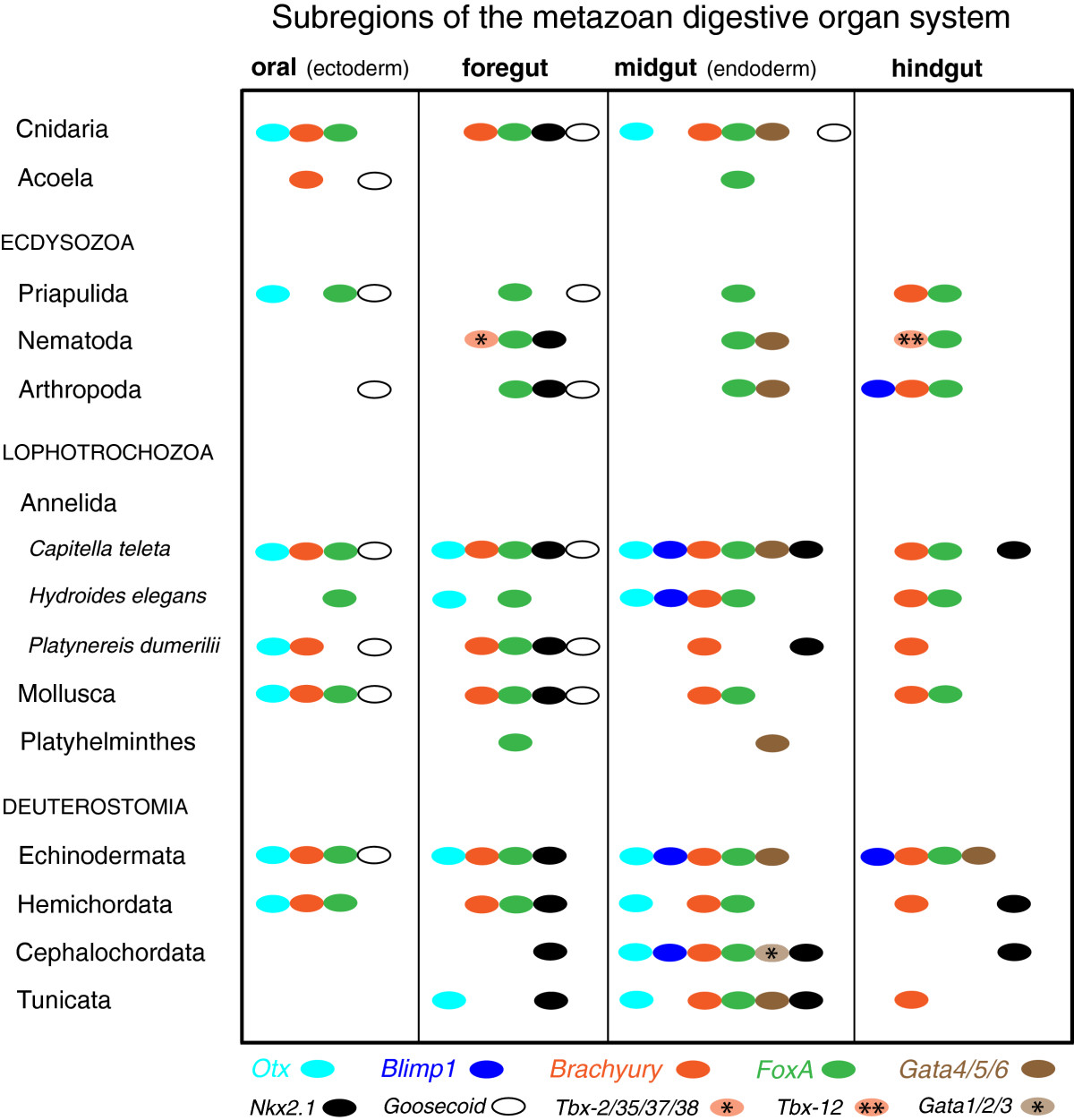
**Comparative gene expression within digestive organ systems of the Metazoa.** Gene orthologs of five endomesoderm transcription factors (*Otx, Blimp1, Brachyury, FoxA, Gata4/5/6*) are designated by colored ovals; additional transcription factors (*Nkx2.1*, *Goosecoid*) are designated by black and white ovals, respectively. Each oval represents gene expression by *in situ* hybridization. For each taxon, gene-specific expression was confirmed in one or more subregions along the digestive organ system [see Additional file9: Table S1]. ‘oral (ectoderm)’ is the future site of mouth formation, or a definitive stomodeum; ‘foregut’ is internal to the mouth opening or described as the foregut, pharynx or esophagus; ‘midgut (endoderm)’ represents endoderm precursor cells in an embryo or gastrula, or is described as the midgut, endoderm or intestine; ‘hindgut’ is the posterior terminus of the gut system, it may include the anus and is described as hindgut, proctodeum or anus. All gene expression patterns were described during a period of development that included gut formation. Where gene-specific expression in a particular gut subregion(s) was detected, it has been indicated in the table. Where no gene expression data are listed for a gene, or gut subregion, then either no expression was detected in that subregion, or expression data do not exist for that particular gene in that taxon. Expression of *Nk2.1* in *C. teleta* is for the *Ct-nkx2.1a* ortholog. A supplementary table and list of references for all expression data in Figure 5 are available in Additional file9: Table S1.

The two most thoroughly described endomesoderm specification networks both utilize interacting transcription factors to specify endomesoderm within the embryo, followed by the specification of distinct mesoderm and endoderm cell lineages in *C. elegans*[103] and the sea urchin[8]. However, they differ in important ways. First, a single endomesoderm progenitor cell (EMS) is specified in the 4-cell embryo of *C. elegans*[104], whereas an endomesoderm territory is specified within micromeres on the vegetal pole in late cleavage-stage embryos of the sea urchin[44]. Second, they differ in which ‘core’ transcription factors are deployed. In *C. elegans*, two pairs of *GATA* factors (*med-1*, *med-2*; *end-1*, *end-3*) are required for establishing the EMS and E cells, and orthologs of *T-box* (*tbx-35*) and *FoxA* (*pha-4*) genes specify the MS blastomere and pharyngeal organ, respectively[20, 105–107]. In the sea urchin, all five ‘kernel’ genes are deployed within the endomesoderm domain[8, 10] and have critical regulatory roles during endoderm specification[11, 13]. Interestingly, we find that in *C. teleta* the expression patterns of *Ct-otx*, *Ct-blimp1* and *Ct-bra* (this study) along with Ct-*foxA* and Ct-*GATA* factors[37], suggest that all five loci may have key roles during development of the endoderm (Figure 4). This common pattern of gene expression between the sea urchin and *C. teleta* implies a remarkable level of similarity in endoderm specification between deuterostomes and lophotrochozoans, which is comparable for only a subset of genes (*FoxA*, *GATA*) in ecdysozoans (Figure 5). And with the exception of *Blimp1* expression, four of the five ‘kernel’ genes are expressed in the endoderm of a cnidarian as well (Figure 5). In subregions outside of endoderm, lophotrochozoans, deuterostomes and cnidarians typically express *Otx*, *Brachyury* and *FoxA* in oral ectoderm and the developing foregut, representing another common pattern of gene expression, although a pattern that has not yet been described for ecdysozoan taxa. When considering individual transcription factors, there are broader trends in gut-related gene expression, including the expression of *Otx* and *FoxA* in oral ectoderm; the expression of *FoxA* in foregut, midgut and hindgut territories; at least one *Gata4/5/6* gene in the midgut; and the expression *FoxA* and *Brachyury* within the hindgut (Figure 5). Across Metazoa, these genes appear to be consistently expressed in distinct subregions (for example, foregut, midgut, hindgut) regardless of germ layer boundaries, suggesting an additional level of molecular similarity underlying gut formation that is uncoupled from embryonic germ layer origin (Figure 5; Additional file9: Table S1 and References).

When compared with sea urchin endomesoderm genes, the expression patterns of either individual or pairs of gene orthologs have been characterized in very few protostome taxa. Because of this, our ability to detect evidence of similar or divergent gene regulatory networks across Metazoa is limited, especially among spiralian taxa. This is particularly surprising when we consider that one of the hallmarks of spiralian development is the highly conserved fate of the 4d cell as a mesendoderm precursor (see[108]). Within Spiralia, *Otx* is expressed in the stomodeum of both the marine polychaete *Platynereis dumerilii* and the limpet *Patella vulgata*, whereas expression in mesoderm or endoderm has not been reported for either of these species[55, 68]. A *brachyury* gene, *Pd-bra*, is expressed in larval foregut and hindgut ectoderm and apparently in midgut endoderm of *P. dumerilii*[55]. In *P. vulgata*, a *brachyury* ortholog (*PvuBra*) is expressed in the 3D cell and other macromeres and in the mesentoblast (4d), which are endodermal and mesodermal precursors, respectively. *PvuBra* is also detected in cells that form parts of the mouth and anus[67]. In the developing trochophore larva of *P. dumerilii*, a single GATA factor (*Pd-GATA456*) is expressed in mesoderm that is likely derived from endomesoderm; however, it is not expressed in endoderm[71]. The expression patterns for *Pd-bra* and *Pd-GATA456* have not yet been traced back to blastomere identities in early embryonic stages when initial germ layer specification is thought to occur in spiralians, although the suggested expression domains are consistent with cell lineage data[109]. A *foxA* gene (*forkhead*) is clearly expressed in the endoderm and larval foregut of *P. vulgata*[56]; in the foregut and hindgut, but not midgut, in another mollusk, *Haliotus rufescens*[50]; in the foregut of *P. dumerilii*[73]; in the pharynx of a turbellarian flatworm[110]; and in presumptive gut tissue in a bryozoan[60]. When compared with *C. teleta*, it appears that only orthologs of *brachyury* and *FoxA* in both *P. dumerilii* and *P. vulgata* show overall similar expression patterns. Prior to this study, expression patterns for *Otx, Blimp1, Bra and FoxA* were characterized in another spiralian, the polychaete *H. elegans*[69, 70, 72, 75], where each ortholog was shown to have some gut-related expression (Figure 5). Those efforts, along with our investigations, are based on an assertion that the ‘kernel’ is considered to be the most ‘evolutionarily inflexible’ component of the endomesoderm GRN[10]. Thus, in a few spiralian taxa, several transcription factors show a conserved pattern of expression relative to the sea urchin endomesoderm ‘kernel’; yet, they also show unique expression domains that are not directly comparable with orthologs in either echinoderms or *C. elegans* (Figure 5).

In addition to our findings that several of the core ‘kernel’ genes show conserved patterns of expression between the sea urchin, *C. teleta,* and other spiralians, there is evidence that additional genes might be conserved within an ancient endomesodermal network. For example, in *C. teleta* it has been shown that in larval stages, *hedgehog*[33] and *Wnt16*[111] are expressed in foregut and hindgut domains, and both *notch* and *delta*[112] are expressed in complex patterns that include foregut tissues. Of even greater interest for comparison with the sea urchin endomesoderm GRN, two *Eve* genes of *C. teleta* (*Ct-eve1* and *Ct-eve2*) are not only expressed in larval foregut and hindgut domains, but are also expressed in mesodermal precursor cells[98], which are typically a source of endomesoderm derived from the mesentoblast (4d) in other spiralians[2, 26]. Furthermore, *Ct-eve1* is expressed in the endoderm of postgastrula stage embryos, in a similar place and time as a *snail* gene (*CapI-sna1*) in *C. teleta*[35]. Both genes may be involved in a regulatory process that controls cell division, shape and differentiation in the presumptive midgut where both *Ct-eve1* and *Cap-sna1* transcripts overlap the endodermal expression domains of *Ct-otx, Ct-blimp1, Ct-bra, Ct-foxA, Ct-gataB1* and *Ct-nkx2.1a*.

## Conclusions

Within Spiralia, data from *Capitella teleta* provide the most comprehensive catalogue of comparable expression patterns for transcriptional regulators of the sea urchin endomesoderm ‘kernel’. Those patterns indicate that five core orthologs of the endomesoderm GRN are involved in regulating endoderm specification and midgut development in *C. teleta*. All five orthologs are also expressed in patterns consistent with roles in foregut development, and a subset of these and other genes are most likely involved with mouth (*Ct-otx, Ct-bra, Ct-foxA, Ct-foxAB* and *Ct-gsc*) and hindgut (*Ct-bra, Ct-foxA, Ct-nkx2.1a* and *Ct-nkx2.1b*) development in *C. teleta*. And by extending the comparison to a broad diversity of animals, we recognize that several of these transcription factors exhibit highly similar patterns of expression in specific gut subregions, both within and outside of the Bilateria (Figure 5). Collectively, those patterns, along with detailed expression patterns presented in this study, indicate there is strong evidence for molecular conservation during metazoan gut formation. Thus, it is likely that networks of ‘gut genes’ were established to regulate development within distinct subregions of animal digestive systems prior to the radiation of Bilateria, with subsequent loss of expression and/or alternate patterns of expression evolving within lineages of Cnidaria, Ecdysozoa, Lophotrochozoa and Deuterostomia. Davidson and Erwin[10] predicted that once comparative network data are available from other animals, ‘there will be found conserved network kernels similar in complexity and character’ to what is characterized in the endomesoderm GRN that is common to a sea urchin and starfish. Although our study of gene expression in *C. teleta* is not a direct indicator of gene function or gene interaction, it is an important resource for comparisons of molecular development within Metazoa, and it provides a tractable and testable target for future functional studies. The next steps for inferring regulatory interactions and establishing the first basic ‘wiring diagrams’ of a network in Spiralia will require quantitative comparisons of the timing and amount of gene expression, and perturbation experiments that interrupt gene function. With the combination of genomic data, gene expression, and a comprehensive cell lineage and fate map of early development, *C. teleta* is emerging as an important research animal for studies on developmental gene regulation.

## Electronic supplementary material

Additional file 1: Document S1: Accession numbers for amino acid sequences used in gene orthology analyses. (PDF 71 KB)

Additional file 2: Figure S1: Orthology analysis of the Otx transcription factor of *Capitella teleta*. The cladogram of the Bayesian consensus tree was produced from an amino-acid alignment of conserved homeodomain regions. The *orthodenticle* gene of *C. teleta* (*Ct-otx*) is a member of the paired class of homeodomain transcription factors, and groups with Otx factors separately from other paired-like homeodomain proteins. Posterior probabilities >0.80 are placed above nodes; maximum likelihood bootstrap values >50% are placed below nodes; there is general agreement between tree topologies. Species abbreviations: Aj, *Apostichopus japonicus*; Ami, *Acropora millepora*; Amphi, *Branchiostoma floridae*; Ci, *Ciona intestinalis*; Cr, *Convolutriloba retrogemma*; Ct, *Capitella teleta*; Dm, *Drosophila melanogaster*; Dr, *Danio rerio*; He, *Hydroides elegans*; Hec, *Herdmania curvata*; Hpr, *Holopneustes purpurescens*; Hs, *Homo sapiens*; Lg, *Lottia gigantea*; Mm, *Mus musculus*; Mmul, *Macaca mulatta*; Nv, *Nematostella vectensis*; Ob, *Octopus bimaculoides*; Pdu, *Platynereis dumerilii*; Pv, *Patella vulgata*; Sko, *Saccoglossus kowalevskii*; Sp, *Strongylocentrotus purpuratus*; Tc, *Tribolium castaneum*. (PDF 109 KB)

Additional file 3: Figure S2: Orthology analysis of the Blimp1 transcription factor of *Capitella teleta*. The cladogram of the Bayesian consensus tree was produced from an amino-acid alignment of conserved C2H2 zinc finger domains. The PRDM-1/B lymphocyte-induced maturation protein-1 gene of *C. teleta* (*Ct-blimp1*) contains both a conserved positive regulatory domain-1 element and zinc finger domain, and groups within a clade of similar proteins. Posterior probabilities >0.80 are placed above nodes; maximum likelihood bootstrap values >50% are placed below nodes; there is general agreement between tree topologies. Species abbreviations: Ag, *Anopheles gambiae* str. PEST; Bf, *Branchiostoma floridae*; Cb, *Caenorhabditis briggsae*; Ce, *Caenorhabditis elegans*; Ct, *Capitella teleta*; Dm, *Drosophila melanogaster*; Dr, *Danio rerio*; Hs, *Homo sapiens*; Lg, *Lottia gigantea*; Mm, *Mus musculus*; Mmul, *Macaca mulatta*; Sp, *Strongylocentrotus purpuratus*; Tr, *Takifugu rubripes*; Tc, *Tribolium castaneum*; X, *Xenopus laevis*. (PDF 99 KB)

Additional file 4: Figure S3: Orthology analysis of the Brachyury transcription factor of *Capitella teleta*. The cladogram of the Bayesian consensus tree was produced from an amino-acid alignment of conserved T-domains. The *brachyury* gene of *C. teleta* (*Ct-bra*) groups within the Brachyury/T subfamily of T-box family DNA-binding proteins. Posterior probabilities >0.80 are placed above nodes; maximum likelihood bootstrap values >50% are placed below nodes; there is general agreement between tree topologies. Species abbreviations: Bf, *Branchiostoma floridae*; Ci, *Ciona intestinalis*; Cl, *Convolutriloba longifissura*; Ct, *Capitella teleta*; Dm, *Drosophila melanogaster*; Lv, *Lytechinus variegatus*; Ml, *Mnemiopsis leydyi*; Mm, *Mus musculus*; Nv, *Nematostella vectensis*; Pd, *Platynereis dumerilii*; Pv, *Patella vulgata*, *Strongylocentrotus purpuratus*; Sk, *Saccostrea kegaki*; Sko, Saccoglossus kowalevskii; Ta *Trichoplax adhaerens*; Tc, *Tribolium castaneum*; X, *Xenopus laevis*. (PDF 100 KB)

Additional file 5: Figure S4: Orthology analysis of the Nkx2.1a transcription factor of *Capitella teleta*. The cladogram of the Bayesian consensus tree was produced from an amino-acid alignment of conserved NKX homeodomains. There are two Nkx2.1 paralogs in *C. teleta*. The *Ct-nkx2.1a* gene groups within a clade of Nkx2.1 proteins that is separate from a clade of Nkx2.2 proteins. Posterior probabilities >0.80 are placed above nodes; maximum likelihood bootstrap values >50% are placed below nodes; there is general agreement between tree topologies. Species abbreviations: Bf, *Branchiostoma floridae*; Ct, *Capitella teleta*; Dm, *Drosophila melanogaster*; Hs, *Homo sapiens*; Lg, *Lottia gigantea*; Pd, *Platynereis dumerilii*; *purpuratus*. (PDF 104 KB)

Additional file 6: Figure S5: Orthology analysis of the Goosecoid transcription factor of *Capitella teleta*. The cladogram of the Bayesian consensus tree was produced from an amino-acid alignment of conserved homeodomains from the paired (PRD) homeobox family of proteins. The *goosecoid* gene of C. teleta (Ct-gsc) groups within a clade of Gsc homeobox proteins. Posterior probabilities >0.80 are placed above nodes; maximum likelihood bootstrap values >50% are placed below nodes; there is general agreement between tree topologies. Species abbreviations: Am, *Apis mellifera*; Bf, *Branchiostoma floridae*; CapI, *Capitella* sp. I (currently known as *C. teleta*); Ct, *Capitella teleta*; Cl, *Convolutriloba longifissura*; Dm, *Drosophila melanogaster*; Dr, *Danio rerio*; Ht, *Heliocidaris tuberculata*; Hv, *Hydra vulgaris*; Lv, *Lytechinus variegatus*; Mm, *Mus musculus*; Nv, *Nematostella vectensis*; Pd, *Platynereis dumerilii*; Pv, *Patella vulgata*, *Strongylocentrotus purpuratus*; Tc, *Tribolium castaneum*. (PDF 100 KB)

Additional file 7: Figure S6: Additional expression patterns of *Otx*, *Blimp1*, *Brachyury* and *FoxAB* in *Capitella teleta*. **(A-B)** Stage 3 mid-gastrula in vegetal (A) and lateral view with vegetal side down (B). *Ct-otx* is expressed in surface and subsurface cells (black arrows) around the posterior side of the blastopore (yellow dashed line), and in cells at the anterior (white arrowheads) and posterior (black arrowheads) sides of the embryo. **(C-D)** Stage 3 gastrula in vegetal (C) and lateral view with vegetal side down (D). *Ct-blimp1* expression is restricted to cells on the vegetal hemisphere (black arrows) within and around the blastopore (yellow dashed line). **(E)** Stage 4 early larva with *Ct-bra* expression in the brain (white arrowhead), stomodeum (white arrow), endoderm (black arrows), and the posterior end of the larva (black arrowhead). **(F-G)** Stage 5 larva showing *Ct-blimp1* expression in the brain (white arrowhead), foregut (white arrows) and endoderm (black arrows). **(H)** Stage 6 larva with *Ct-bra* expression in the brain (white arrowheads), foregut (white arrows), mesoderm along ventro-lateral sides of the trunk (dashed arrows), and the anus (black arrowhead). **(I)** Stage 7 larva with *Ct-foxAB* expression in the brain (white arrowheads), mouth (asterisk), foregut (white arrows) and ventro-lateral mesoderm of posterior segments (dashed arrows). Asterisk marks the position of the mouth; anterior is to the left in all panels. Abbreviations: lat, lateral; vent, ventral; veg, vegetal. (PNG 4 MB)

Additional file 8: Figure S7: Expression of *Nkx2.1b* in *Capitella teleta* larvae. **(A-B)** *Ct-nkx2.1b* is expressed in both lobes of the brain (white arrowheads), and in a subsurface domain on either side of the stomodeum (white arrows) in stage 4 larvae. **(C)** Expression of *Ct-nkx2.1b* in brain (white arrowheads), foregut (white arrows) and a ventrolateral domain in the ectoderm (white dashed arrows) and mesoderm (dashed arrows) of the trunk during stage 5. **(D)** In stage 6 larvae, there is expression of *Ct-nkx2.1b* in the brain (white arrowheads), foregut (white arrows), in ectoderm, including in the ventral nerve cord (white dashed arrows), mesoderm of the trunk (dashed arrows), and hindgut (black arrowhead). The image in each panel was created by combining micrographs from a series of focal planes. Asterisk marks the position of the mouth; anterior is to the left in all panels. Abbreviations: lat, lateral; vent, ventral. (PNG 3 MB)

Additional file 9: Table S1: References for gene expression within metazoan digestive organ systems. The data in **Table S1** correspond to the gene expression data (colored, black and white ovals) summarized in Figure 5 of the manuscript. Each number within the table is matched with the corresponding number of its published reference in the **References for Table S1** below the table. In several cases, for a particular taxon (left side of table) and gene (top of table) combination, the expression patterns were compiled from more than one study of the same species, or more than one species within a taxon, and are represented by multiple references. All of the references are for expression data obtained by in situ hybridization. For additional information, see the figure caption for Figure 5. (PDF 191 KB)

## Abbreviations

BS: bootstrap
GRN: gene regulatory network
PP: posterior probability.
