# Supplementary material for: Molecular conservation of metazoan gut formation: evidence from expression of endomesoderm genes in Capitella teleta (Annelida)
Source: EvoDevo. 2014 Oct 29;5:39. doi: 10.1186/2041-9139-5-39 (PMC4407770; doi:10.1186/2041-9139-5-39)
Supplement: Supplementary file 1 — Additional file 1: Document S1: Accession numbers for amino acid sequences used in gene orthology analyses. (PDF 71 KB) [file 13227_2014_134_MOESM1_ESM.pdf]

Additional file 6: Document S1

Accession numbers for amino acid sequences used in gene orthology analyses.

Otx (Orthodenticle)

[AmiOtxA, GenBank: ABK41270; AmiOtxB, GenBank: ABK41271; AjOtx, GenBank: BAB16104; AmphiOtx, GenBank: AAC00193; Ci-Otx, GenBank: NP_001027662; CrOtx1, GenBank: ACT22574; CrOtx2, GenBank: ACT22575; DmOtd, GenBank: CAA41732; Hec-Otx, GenBank: AAD30504; HprOtx, GenBank: AAQ24027; HeOtx, GenBank: ABK76302; Lg194184, JGI: 194184; MmOTX2, GenBank: NP_569090; MmOTX1, GenBank: P80205; NvOtxA, GenBank:ACO53861; NvOtxB, , GenBank: NvOtxB, GenBank:ACO53862; NvOtxC, GenBank: ACO53863; ObOtxl, GenBank: AZ99218; Pv-Otx, GenBank: AAM33144; Pdu-Otx, GenBank: UPI0000082184; SkoOtx, GenBank: NP_001158360; SpOtxβ, GenBank: NP_001027540; SpOTX, GenBank: Q26417; TcOtd-1, GenBank: NP_001034513; TcOtd-2, GenBank: NP_001034526;]

Outgroups: [Ct-Pax3/7, GenBank: ABC68267; DmPrd, GenBank: NP_723721; MmPax3, GenBank: P24610; MmPax7, GenBank: NP_03516]

Blimp1 (B-lymphocyte-inducing maturation protein-1)

[AgamP006592-PA, GenBank: XP_316619; AmphiBlimp1, GenBank: ACH72078; CBR-Blmp-1, GenBank: XP_00263975; CeBlmp1, GenBank: NP_492723; DrBlimp-1, GenBank: NP_955809; DmBlimp-1, GenBank: NP_647982; HsPRDM1α, GenBank: NP_001189; HsPRDM1β, GenBank: NP_878911; Lg86198, JGI: 86198; MmulPRDM1, GenBank: XP_001087708; MmPRDM1, GenBank: NP_031574; SpBlimp1/Krox, GenBank: NP_001073021; FuguBlimp-1, GenBank: NP_001027861; TcBlimp1, GenBank: EFA04698; Xblimp1, GenBank: AAF08791]

Outgroups: [Ct-168004, JGI: 168004; MmSCAN-KRAB, GenBank: AAG00602; MmPRDM4, GenBank: NP_857633; SpKrl, GenBank: AG31160]

Bra (Brachyury)

[AmphiBra-1, GenBank: Q17134; AmphiBra-2, GenBank: P80492; Ci-Bra, GenBank: NP_001027659; ClBra,GenBank: ACG70808; DmByn, GenBank: NP_524031; LvBra, GenBank: AAL27986; MlBra,GenBank: ABL68078; MmBra, GenBank: NP_033335; NvBra1, GenBank: AAO27886; Pv-Bra,GenBank: CAD12821; Pdu-Bra, GenBank: CAC19335; SkoBra, GenBank: NP_001158367; SkBra, GenBank: BAG68616; TcBra,GenBank: NP_001034532; TaBra, GenBank: CAD70269; Xbra, GenBank: P24781; Xbrab,GenBank: NP_001085165]

Outgroups: [Ct-152028, JGI: 152028; Ct-168974, JGI: 168974; Ct-223644, JGI: 223644; DmBi, GenBank: NP_525070; DmH15, GenBank: NP_608926; MmTBX2, GenBank: NP_033350; MmTBX20, GenBank: Q9ES03; MmTbr1, GenBank: NP_033348]

Nk2.1a (NK Family)

[AmphiNk2-1, GenBank: XP_002589194; AmphiNk2-2, GenBank: AAD01958; AmphiNk3, GenBank: XP_002610434; Ct-NKx2.1a, GenBank: ACH89430; Ct-NKx2.1b, GenBank: ACH89431; Ct-NKx2.2a, GenBank: ACH89432; Ct-NKx2.2b, GenBank: ACH89433; Ct-NKx3, GenBank: ACI26670; Ct-NKx4a, GenBank: ACH89434; Ct-NKx4b, GenBank: ACH89435; DmBap, GenBank: NP_732637; DmScro, GenBank: NP_001015473; DmTin, GenBank: NP_524433; DmVnd, GenBank: NP_476786; HsNkx-2.1, GenBank: NP_001073136; HsNkx-2.2, GenBank: NP_002500; HsNkx-2.3, GenBank: NP_660328; HsNkx-2.4, GenBank: CAH71494; HsNkx-2.5, GenBank: NP_004378; HsNkx-2.6, GenBank: NP_001129743; HsNkx-2.8, GenBank: NP_055175; HsNkx-3.1, GenBank: NP_006158; HsNkx-3.2, GenBank: NP_001180; Lg161410, JGI: 161410; Lg123727, JGI: 123727; Lg231718, JGI: 231718; Lg178221, JGI: 178221; Pdu-Nk2.1, GenBank: CAJ38809; Pdu-NK3, GenBank: ABQ10641; Pdu-NK4, GenBank: ABQ10640]

Gsc (Goosecoid)

[AmGscl, GenBank: XP_01121922; AmphiGsc, GenBank: AAF97935; ClGsc, GenBank: ACG70804; DrGsc, GenBank: NP_571092; DmGsc, GenBank: P54366; HtGsc, GenBank: AAY86176; Cngsc, GenBank: AAF14575; Lg107589, JGI: 107589; LvGsc, GenBank: AAR17089; MmGsc,GenBank: NP_034481; MmGsc2, GenBank: NP_083745; NvGscl, GenBank: ABA46370; Pv-Gsc, GenBank: CAD45551; Pdu-Gsc, GenBank: CAC19336; SkoGsc, GenBank: NP_00116156; SpGsc, GenBank: NP_999663; TcGsc, GenBank: EFA10784]

Outgroups: [Ct-99648, JGI: 99648; CapI-Hbnl, GenBank: ABC58683; DmOtp, GenBank: P56672; DmHbn, GenBank: CAC06429; DmEy, GenBank: NP_524628; MmOtp, GenBank: NP_035151; MmPax4, GenBank: NP_035168; MmPax6, GenBank: NP_038655]
