# Supplementary figures and images for: Molecular conservation of metazoan gut formation: evidence from expression of endomesoderm genes in Capitella teleta (Annelida)
Source: EvoDevo. 2014 Oct 29;5:39. doi: 10.1186/2041-9139-5-39 (PMC4407770; doi:10.1186/2041-9139-5-39)

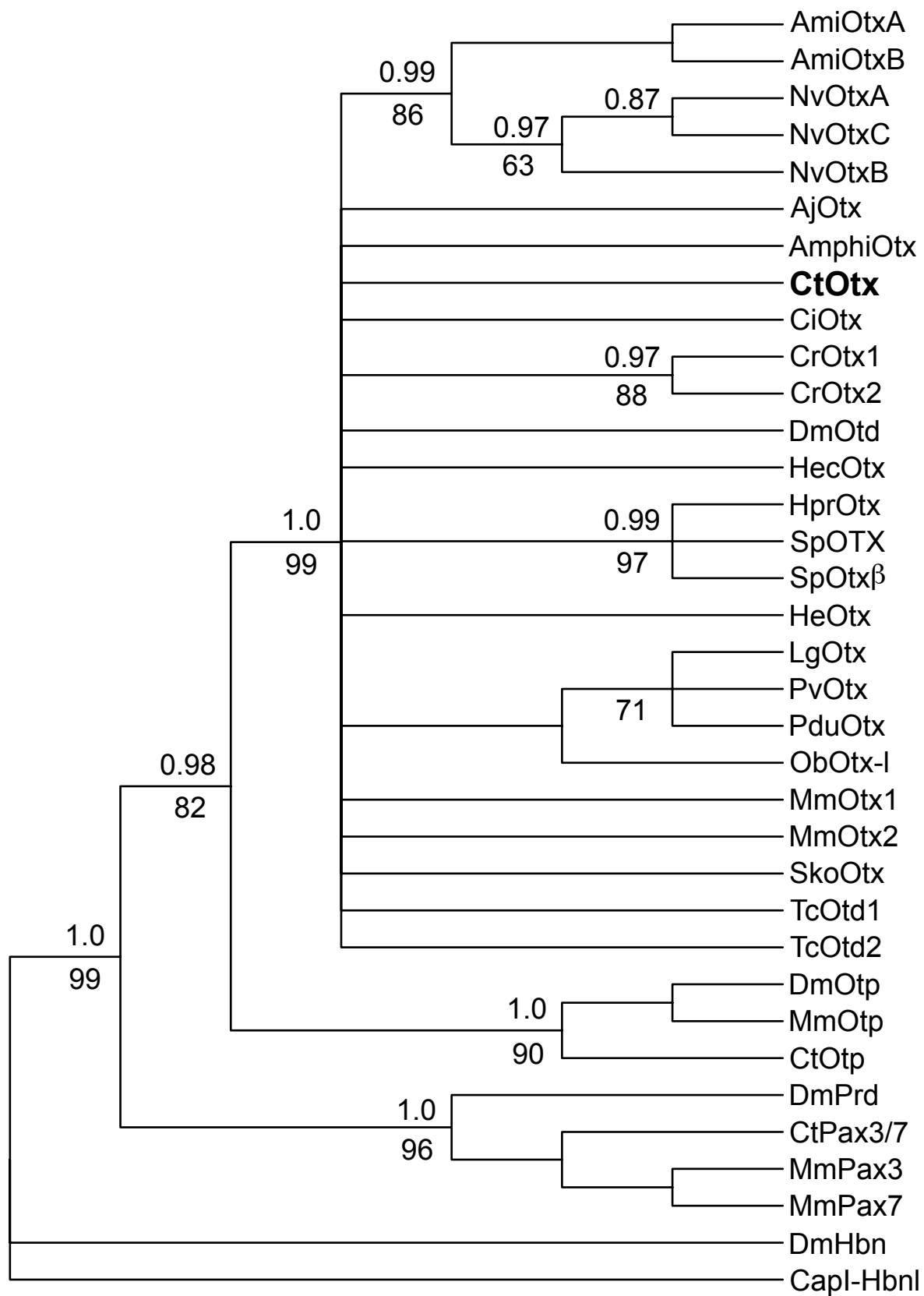

Supplement: Supplementary file 2 — Additional file 2: Figure S1: Orthology analysis of the Otx transcription factor of Capitella teleta. The cladogram of the Bayesian consensus tree was produced from an amino-acid alignment of conserved homeodomain regions. The orthodenticle gene of C. teleta (Ct-otx) is a member of the paired class of homeodomain transcription factors, and groups with Otx factors separately from other paired-like homeodomain proteins. Posterior probabilities >0.80 are placed above nodes; maximum likelihood bootstrap values >50% are placed below nodes; there is general agreement between tree topologies. Species abbreviations: Aj, Apostichopus japonicus; Ami, Acropora millepora; Amphi, Branchiostoma floridae; Ci, Ciona intestinalis; Cr, Convolutriloba retrogemma; Ct, Capitella teleta; Dm, Drosophila melanogaster; Dr, Danio rerio; He, Hydroides elegans; Hec, Herdmania curvata; Hpr, Holopneustes purpurescens; Hs, Homo sapiens; Lg, Lottia gigantea; Mm, Mus musculus; Mmul, Macaca mulatta; Nv, Nematostella vectensis; Ob, Octopus bimaculoides; Pdu, Platynereis dumerilii; Pv, Patella vulgata; Sko, Saccoglossus kowalevskii; Sp, Strongylocentrotus purpuratus; Tc, Tribolium castaneum. (PDF 109 KB) [file 13227_2014_134_MOESM2_ESM.pdf]

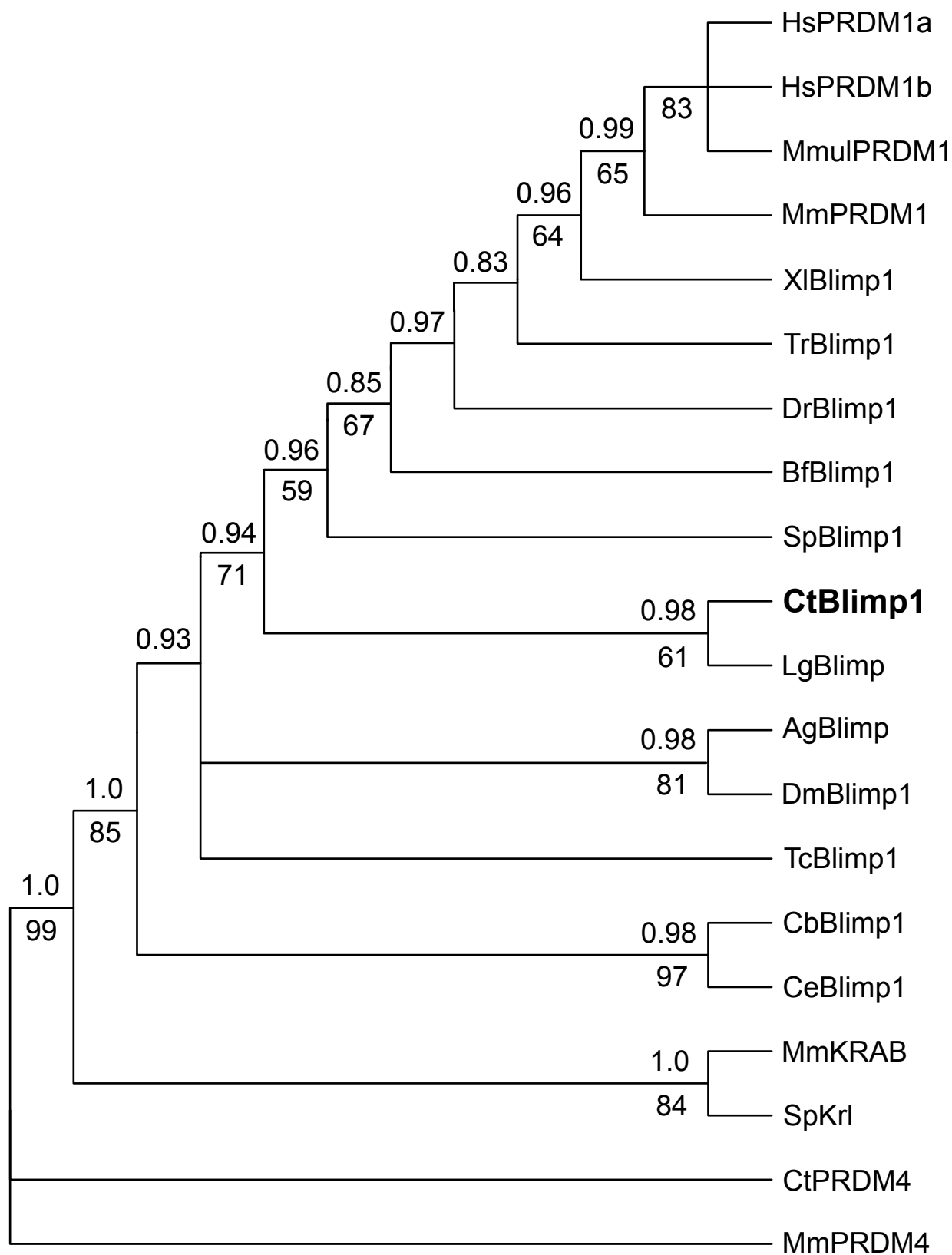

Supplement: Supplementary file 3 — Additional file 3: Figure S2: Orthology analysis of the Blimp1 transcription factor of Capitella teleta. The cladogram of the Bayesian consensus tree was produced from an amino-acid alignment of conserved C2H2 zinc finger domains. The PRDM-1/B lymphocyte-induced maturation protein-1 gene of C. teleta (Ct-blimp1) contains both a conserved positive regulatory domain-1 element and zinc finger domain, and groups within a clade of similar proteins. Posterior probabilities >0.80 are placed above nodes; maximum likelihood bootstrap values >50% are placed below nodes; there is general agreement between tree topologies. Species abbreviations: Ag, Anopheles gambiae str. PEST; Bf, Branchiostoma floridae; Cb, Caenorhabditis briggsae; Ce, Caenorhabditis elegans; Ct, Capitella teleta; Dm, Drosophila melanogaster; Dr, Danio rerio; Hs, Homo sapiens; Lg, Lottia gigantea; Mm, Mus musculus; Mmul, Macaca mulatta; Sp, Strongylocentrotus purpuratus; Tr, Takifugu rubripes; Tc, Tribolium castaneum; X, Xenopus laevis. (PDF 99 KB) [file 13227_2014_134_MOESM3_ESM.pdf]

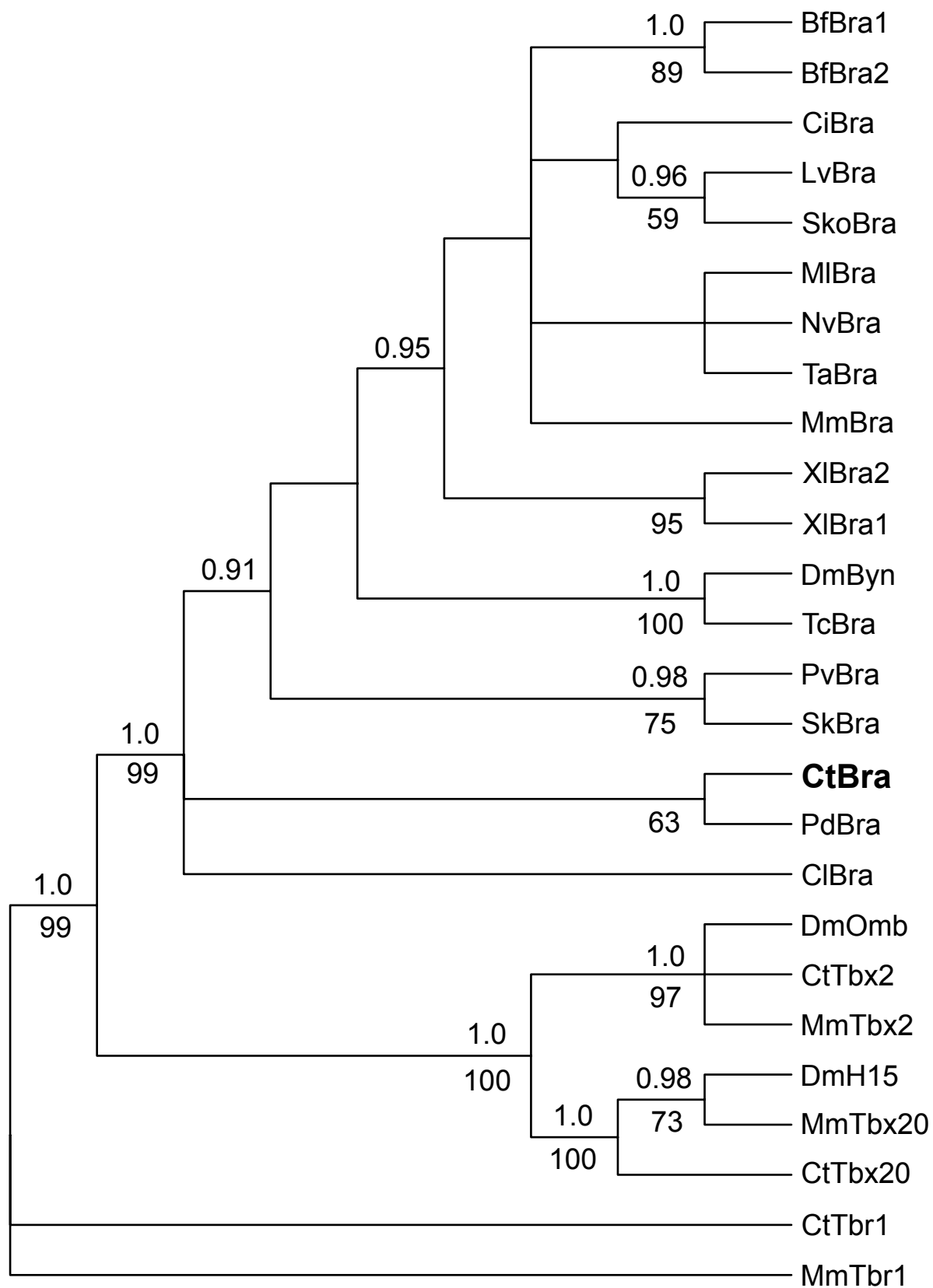

Supplement: Supplementary file 4 — Additional file 4: Figure S3: Orthology analysis of the Brachyury transcription factor of Capitella teleta. The cladogram of the Bayesian consensus tree was produced from an amino-acid alignment of conserved T-domains. The brachyury gene of C. teleta (Ct-bra) groups within the Brachyury/T subfamily of T-box family DNA-binding proteins. Posterior probabilities >0.80 are placed above nodes; maximum likelihood bootstrap values >50% are placed below nodes; there is general agreement between tree topologies. Species abbreviations: Bf, Branchiostoma floridae; Ci, Ciona intestinalis; Cl, Convolutriloba longifissura; Ct, Capitella teleta; Dm, Drosophila melanogaster; Lv, Lytechinus variegatus; Ml, Mnemiopsis leydyi; Mm, Mus musculus; Nv, Nematostella vectensis; Pd, Platynereis dumerilii; Pv, Patella vulgata, Strongylocentrotus purpuratus; Sk, Saccostrea kegaki; Sko, Saccoglossus kowalevskii; Ta Trichoplax adhaerens; Tc, Tribolium castaneum; X, Xenopus laevis. (PDF 100 KB) [file 13227_2014_134_MOESM4_ESM.pdf]

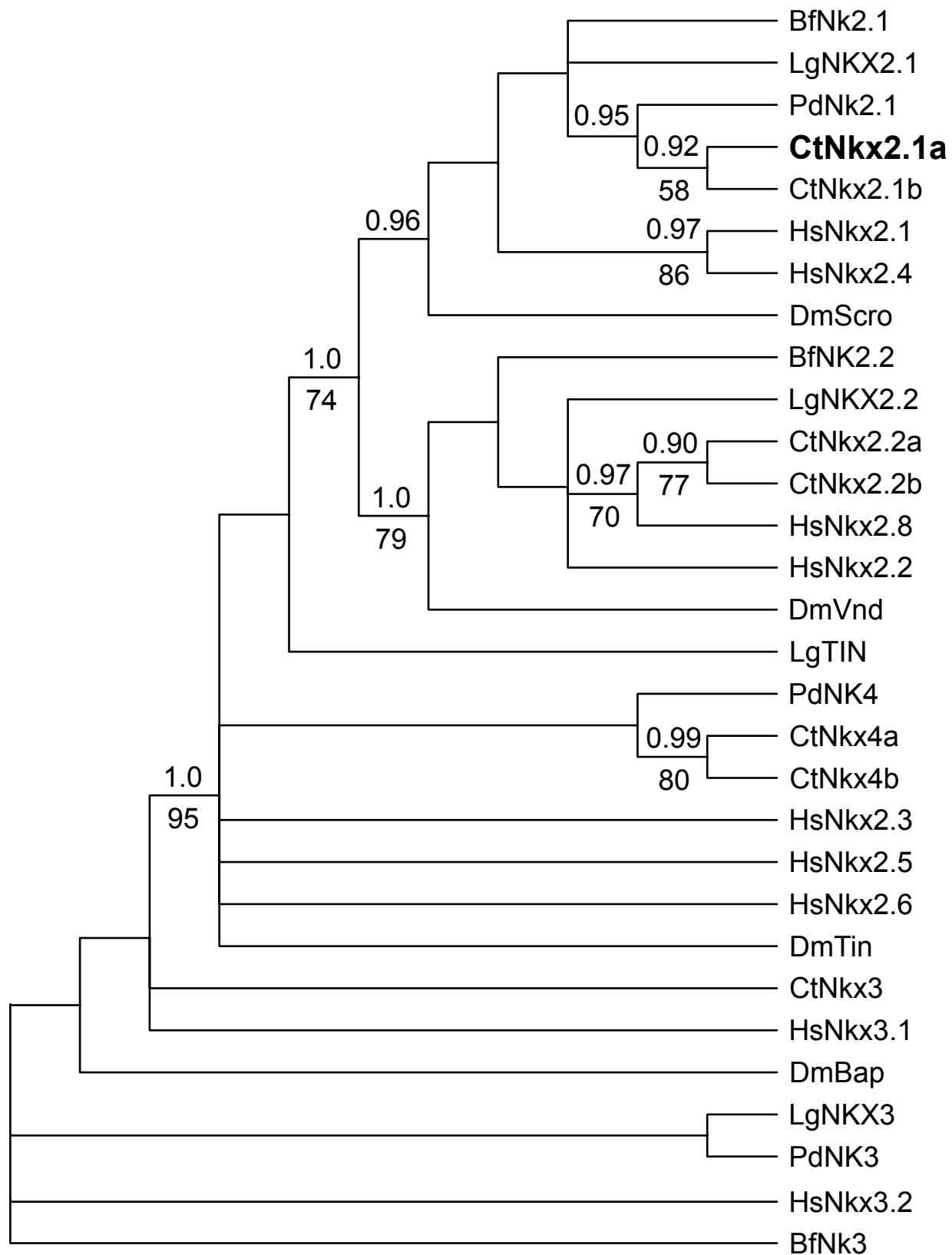

Supplement: Supplementary file 5 — Additional file 5: Figure S4: Orthology analysis of the Nkx2.1a transcription factor of Capitella teleta. The cladogram of the Bayesian consensus tree was produced from an amino-acid alignment of conserved NKX homeodomains. There are two Nkx2.1 paralogs in C. teleta. The Ct-nkx2.1a gene groups within a clade of Nkx2.1 proteins that is separate from a clade of Nkx2.2 proteins. Posterior probabilities >0.80 are placed above nodes; maximum likelihood bootstrap values >50% are placed below nodes; there is general agreement between tree topologies. Species abbreviations: Bf, Branchiostoma floridae; Ct, Capitella teleta; Dm, Drosophila melanogaster; Hs, Homo sapiens; Lg, Lottia gigantea; Pd, Platynereis dumerilii; purpuratus. (PDF 104 KB) [file 13227_2014_134_MOESM5_ESM.pdf]

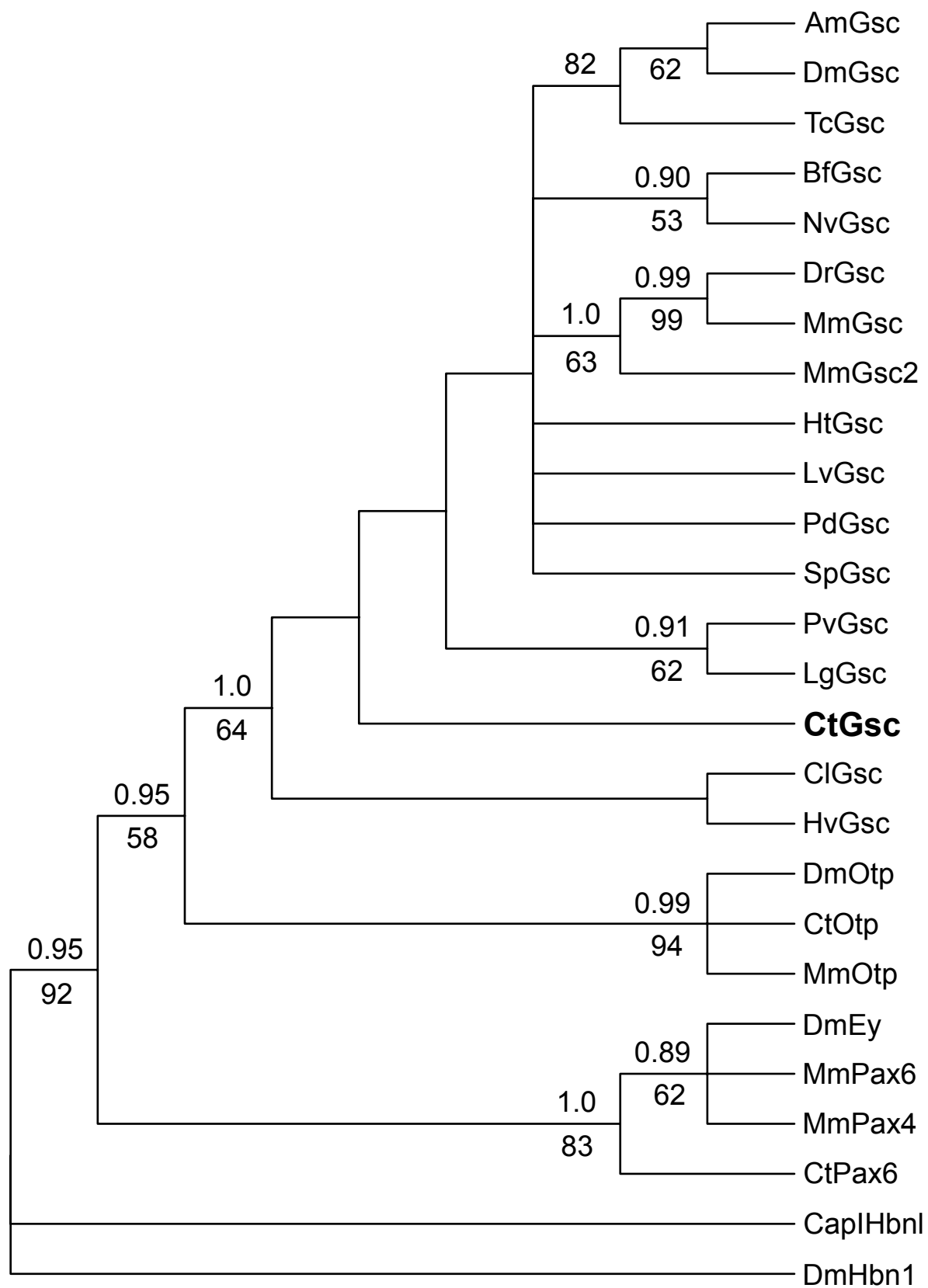

Supplement: Supplementary file 6 — Additional file 6: Figure S5: Orthology analysis of the Goosecoid transcription factor of Capitella teleta. The cladogram of the Bayesian consensus tree was produced from an amino-acid alignment of conserved homeodomains from the paired (PRD) homeobox family of proteins. The goosecoid gene of C. teleta (Ct-gsc) groups within a clade of Gsc homeobox proteins. Posterior probabilities >0.80 are placed above nodes; maximum likelihood bootstrap values >50% are placed below nodes; there is general agreement between tree topologies. Species abbreviations: Am, Apis mellifera; Bf, Branchiostoma floridae; CapI, Capitella sp. I (currently known as C. teleta); Ct, Capitella teleta; Cl, Convolutriloba longifissura; Dm, Drosophila melanogaster; Dr, Danio rerio; Ht, Heliocidaris tuberculata; Hv, Hydra vulgaris; Lv, Lytechinus variegatus; Mm, Mus musculus; Nv, Nematostella vectensis; Pd, Platynereis dumerilii; Pv, Patella vulgata, Strongylocentrotus purpuratus; Tc, Tribolium castaneum. (PDF 100 KB) [file 13227_2014_134_MOESM6_ESM.pdf]

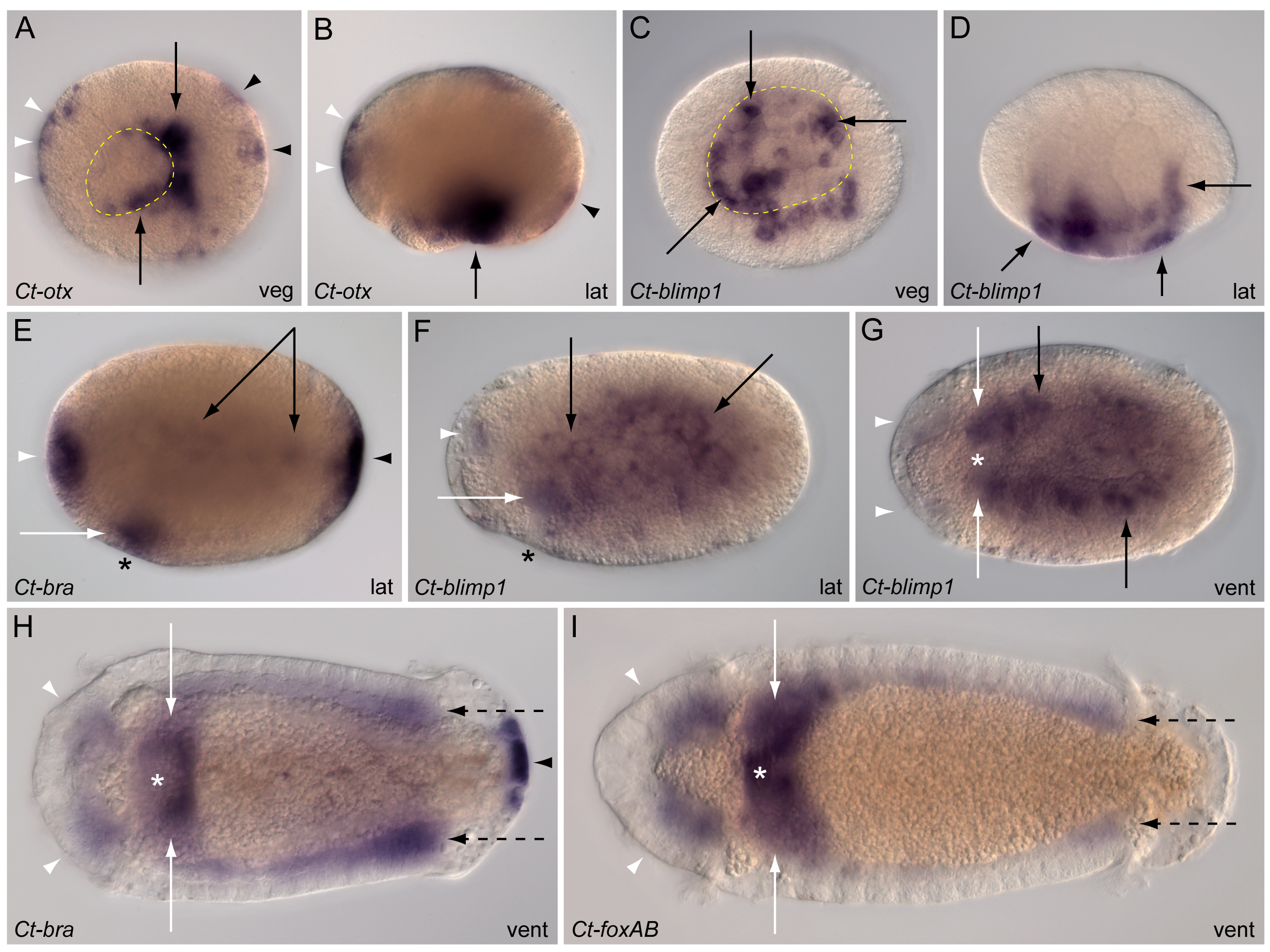

Supplement: Supplementary file 7 — Additional file 7: Figure S6: Additional expression patterns of Otx, Blimp1, Brachyury and FoxAB in Capitella teleta. (A-B) Stage 3 mid-gastrula in vegetal (A) and lateral view with vegetal side down (B). Ct-otx is expressed in surface and subsurface cells (black arrows) around the posterior side of the blastopore (yellow dashed line), and in cells at the anterior (white arrowheads) and posterior (black arrowheads) sides of the embryo. (C-D) Stage 3 gastrula in vegetal (C) and lateral view with vegetal side down (D). Ct-blimp1 expression is restricted to cells on the vegetal hemisphere (black arrows) within and around the blastopore (yellow dashed line). (E) Stage 4 early larva with Ct-bra expression in the brain (white arrowhead), stomodeum (white arrow), endoderm (black arrows), and the posterior end of the larva (black arrowhead). (F-G) Stage 5 larva showing Ct-blimp1 expression in the brain (white arrowhead), foregut (white arrows) and endoderm (black arrows). (H) Stage 6 larva with Ct-bra expression in the brain (white arrowheads), foregut (white arrows), mesoderm along ventro-lateral sides of the trunk (dashed arrows), and the anus (black arrowhead). (I) Stage 7 larva with Ct-foxAB expression in the brain (white arrowheads), mouth (asterisk), foregut (white arrows) and ventro-lateral mesoderm of posterior segments (dashed arrows). Asterisk marks the position of the mouth; anterior is to the left in all panels. Abbreviations: lat, lateral; vent, ventral; veg, vegetal. (PNG 4 MB) [file 13227_2014_134_MOESM7_ESM.png]

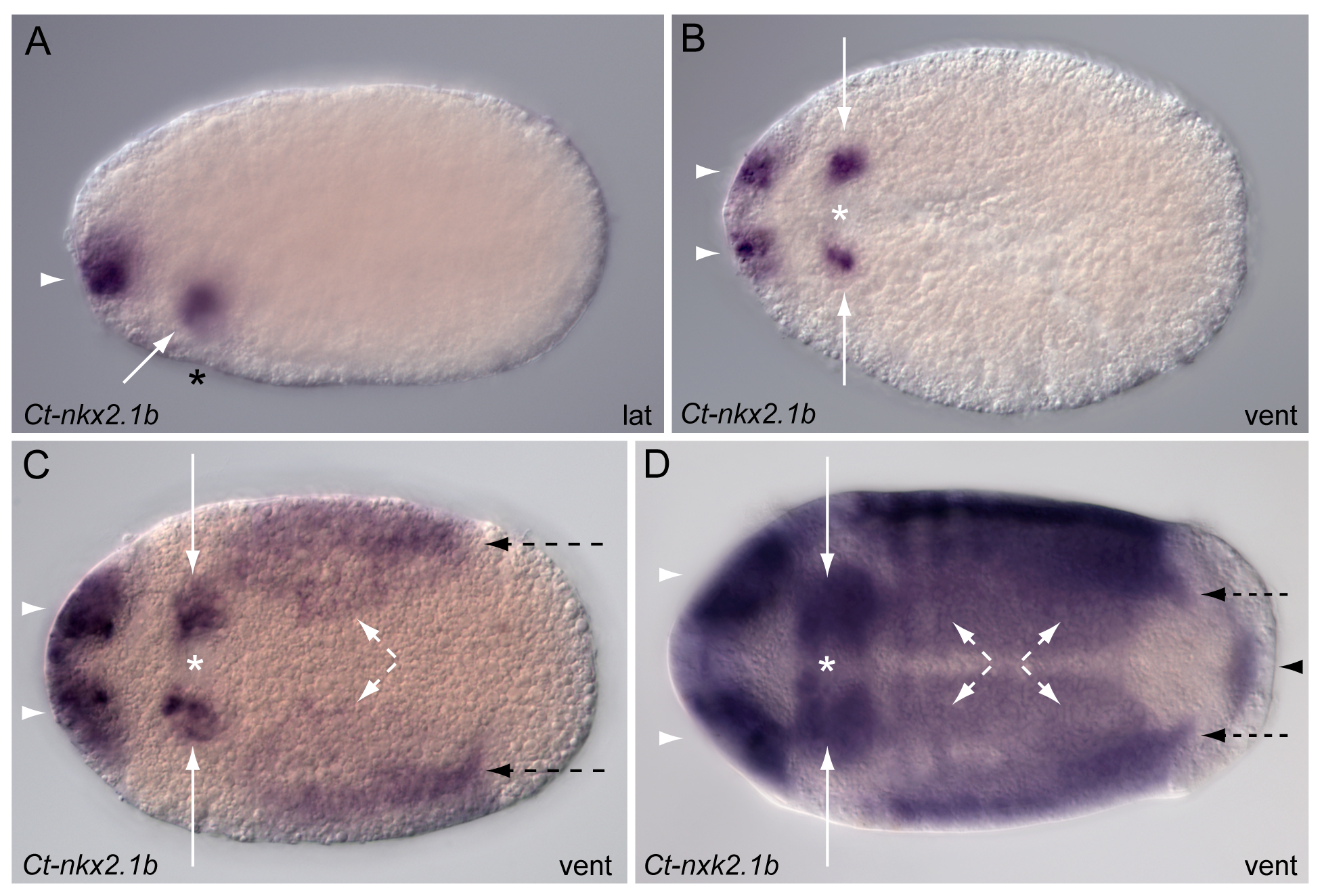

Supplement: Supplementary file 8 — Additional file 8: Figure S7: Expression of Nkx2.1b in Capitella teleta larvae. (A-B) Ct-nkx2.1b is expressed in both lobes of the brain (white arrowheads), and in a subsurface domain on either side of the stomodeum (white arrows) in stage 4 larvae. (C) Expression of Ct-nkx2.1b in brain (white arrowheads), foregut (white arrows) and a ventrolateral domain in the ectoderm (white dashed arrows) and mesoderm (dashed arrows) of the trunk during stage 5. (D) In stage 6 larvae, there is expression of Ct-nkx2.1b in the brain (white arrowheads), foregut (white arrows), in ectoderm, including in the ventral nerve cord (white dashed arrows), mesoderm of the trunk (dashed arrows), and hindgut (black arrowhead). The image in each panel was created by combining micrographs from a series of focal planes. Asterisk marks the position of the mouth; anterior is to the left in all panels. Abbreviations: lat, lateral; vent, ventral. (PNG 3 MB) [file 13227_2014_134_MOESM8_ESM.png]
