# Supplementary material for: Molecular conservation of metazoan gut formation: evidence from expression of endomesoderm genes in Capitella teleta (Annelida)
Source: EvoDevo. 2014 Oct 29;5:39. doi: 10.1186/2041-9139-5-39 (PMC4407770; doi:10.1186/2041-9139-5-39)
Supplement: Supplementary file 9 — Additional file 9: Table S1: References for gene expression within metazoan digestive organ systems. The data in Table S1 correspond to the gene expression data (colored, black and white ovals) summarized in Figure 5 of the manuscript. Each number within the table is matched with the corresponding number of its published reference in the References for Table S1 below the table. In several cases, for a particular taxon (left side of table) and gene (top of table) combination, the expression patterns were compiled from more than one study of the same species, or more than one species within a taxon, and are represented by multiple references. All of the references are for expression data obtained by in situ hybridization. For additional information, see the figure caption for Figure 5. (PDF 191 KB) [file 13227_2014_134_MOESM9_ESM.pdf]

Additonal file 9: Table S1

Table S1. References for expression data in Figure 5: Subregions of the Metazoan Digestive Organ System

	orthodenticle	*blimp1*	*brachyury*	*foxA*	*gata4/5/6*	*nkx2.1*	*goosecoid*
Cnidaria	1		2, 3	4, 5, 6, 7	5	8	9
Acoela			10	10, 11			10
Priapulida	12		12	12			12
Nematoda			13**,14*,15*, 16*	17, 18	19, 20, 21	22	
Arthropoda		23	24, 25	26	27, 28, 29	30	31
Annelida							
Capitella teleta	this study	this study	this study	32	32	this study	this study
Hydroides elegans	33	34	35	36			
Platynereis dumerilii	37		37	38		39	37
Mollusca	40		41	42, 43		43	42
Platyhelminthes				44	45		
Echinodermata	46, 47	48	49, 50	51	52, 53	54, 43	55
Hemichordata	56		57, 58	58, 59		60	
Cephalochordata	61	62	63	64	65***	66	
Tunicata	67, 68, 69		70	71	72, 73	74	

Numbers in columns of this table correspond with numbered references below. * Tbx-2/35/37/38 ** Tbx-12 *** gata1/2/3

References for Table S1

1. Mazza ME, Pang K, Martindale MQ, Finnerty JR: **Genomic organization, gene structure, and developmental expression of three clustered *otx* genes in the sea anemone *Nematostella vectensis*.** *J Exp Zool Part B* 2007, **308:**494-506.
2. Technau U, Bode HR: ***HyBra1*, a *Brachyury* homologue, acts during head formation in *Hydra*.** *Development* 1999, **126:**999-1010.
3. Scholz CB, Technau U: **The ancestral role of *Brachyury*: expression of *NemBra1* in the basal cnidarian *Nematostella vectensis* (Anthozoa).** *Dev Genes Evol* 2003, **212:**563-570.
4. Martinez DE, Dirksen M-L, Bode PM, Jamrich M, Steele RE, Bode HR: ***Budhead*, a Fork Head/HNF-3 Homologue, Is Expressed during Axis Formation and Head Specification in *Hydra*.** *Dev Biol* 1997, **192:**523-536.
5. Martindale MQ, Pang K, Finnerty JR: **Investigating the origins of triploblasty: 'mesodermal' gene expression in a diploblastic animal, the sea anemone *Nematostella vectensis* (phylum, Cnidaria; class, Anthozoa).** *Development* 2004, **131:**2463-2474.

6. Fritzenwanker JH, Saina M, Technau U: **Analysis of *forkhead* and *snail* expression reveals epithelial-mesenchymal transitions during embryonic and larval development of *Nematostella vectensis*.** *Dev Biol* 2004, **275:**389-402.
7. Magie CR, Daly M, Martindale MQ: **Gastrulation in the cnidarian *Nematostella vectensis* occurs via invagination not ingression.** *Dev Biol* 2007, **305:**483-497.
8. Saina M, Genikhovich G, Renfer E, Technau U: **BMPs and Chordin regulate patterning of the directive axis in a sea anemone.** *Proc Natl Acad Sci U S A* 2009, **106:**18592-18597.
9. Matus DQ, Pang K, Marlow H, Dunn CW, Thomsen GH, Martindale MQ: **Molecular evidence for deep evolutionary roots of bilaterality in animal development.** *Proc Natl Acad Sci U S A* 2006, **103:**11195-11200.
10. Hejnol A, Martindale MQ: **Acoel development indicates the independent evolution of the bilaterian mouth and anus.** *Nature* 2008, **456:**382-386.
11. Chiodin M, Borve A, Berezikov E, Ladurner P, Martinez P, Hejnol A: **Mesodermal gene expression in the acoel *Isodiametra pulchra* indicates a low number of mesodermal cell types and the endomesodermal origin of the gonads.** *PloS one* 2013, **8:**e55499.
12. Martin-Duran JM, Janssen R, Wennberg S, Budd GE, Hejnol A: **Deuterostomic development in the protostome *Priapulus caudatus*.** *Curr Biol* 2012, **22:**2161-2166.
13. Woollard A, Hodgkin J: **The *Caenorhabditis elegans* fate-determining gene *mab-9* encodes a T-box protein required to pattern the posterior hindgut.** *Genes Dev* 2000, **14:**596-603.
14. Good K, Ciosk R, Nance J, Neves A, Hill RJ, Priess JR: **The T-box transcription factors TBX-37 and TBX-38 link GLP-1/Notch signaling to mesoderm induction in *C. elegans* embryos.** *Development* 2004, **131:**1967-1978.
15. Broitman-Maduro G, Lin KT, Hung WW, Maduro MF: **Specification of the *C. elegans* MS blastomere by the T-box factor TBX-35.** *Development* 2006, **133:**3097-3106.
16. Chowdhuri SR, Crum T, Woollard A, Aslam S, Okkema PG: **The T-box factor TBX-2 and the SUMO conjugating enzyme UBC-9 are required for ABa-derived pharyngeal muscle in *C. elegans*.** *Dev Biol* 2006, **295:**664-677.
17. Azzaria M, Goszczynski B, Chung MA, Kalb JM, McGhee JD: **A *fork head*/HNF-3 Homolog Expressed in the Pharynx and Intestine of the *Caenorhabditis elegans* Embryo.** *Dev Biol* 1996, **178:**289-303.
18. Kalb JM, Lau KK, Goszczynski B, Fukushige T, Moons D, Okkema PG, McGhee JD: ***pha-4* is *Ce-fkh-1*, a *fork head*/HNF-3α,β,γ homolog that functions in organogenesis of the *C. elegans* pharynx.** *Development* 1998, **125:**2171-2180.
19. Zhu J, Hill RJ, Heid PJ, Fukuyama M, Sugimoto A, Priess JR, Rothman JH: ***end-1* encodes an apparent GATA factor that specifies the endoderm precursor in *Caenorhabditis elegans* embryos.** *Genes Dev* 1997, **11:**2883-2896.
20. Fukushige T, Hawkins MG, McGhee JD: **The GATA-factor *elt-2* is essential for formation of the *Caenorhabditis elegans* intestine.** *Dev Biol* 1998, **198:**286-302.
21. Maduro MF, Rothman JH: **Making worm guts: the gene regulatory network of the *Caenorhabditis elegans* endoderm.** *Dev Biol* 2002, **246:**68-85.
22. Harfe BD, Fire A: **Muscle and nerve-specific regulation of a novel NK-2 class homeodomain factor in *Caenorhabditis elegans*.** *Development* 1998, **125:**421-429.

23. Teclise N, Fengwei Y, Sudipto R: **A homologue of the vertebrate SET domain and zinc finger protein Blimp-1 regulates terminal differentiation of the tracheal system in the *Drosophila* embryo.** *Dev Genes Evol* 2006, **216:**243-252.
24. Kispert A, Herrmann BG, Leptin M, Reuter R: **Homologs of the mouse *Brachyury* gene are involved in the specification of posterior terminal structures in *Drosophila*, *Tribolium*, and *Locusta*.** *Gene Dev* 1994, **8:**2137-2150.
25. Singer JB, Harbecke R, Kusch T, Reuter R, Lengyel JA: ***Drosophila brachyenteron* regulates gene activity and morphogenesis in the gut.** *Development* 1996, **122:**3707-3718.
26. Weigel D, Jurgens G, Kuttner F, Seifert E, Jackle H: **The homeotic gene *fork head* encodes a nuclear protein and is expressed in the terminal regions of the *Drosophila* embryo.** *Cell* 1989, **57:**645-658.
27. Abel T, Michelson AM, Maniatis T: **A *Drosophila* GATA family member that binds to Adh regulatory sequences is expressed in the developing fat body.** *Development* 1993, **119:**623-633.
28. Reuter R: **The gene *serpent* has homeotic properties and specifies endoderm versus ectoderm within the *Drosophila* gut.** *Development* 1994, **120:**1123-1135.
29. Okumura T, Matsumoto A, Tanimura T, Murakami R: **An endoderm-specific GATA factor gene, *dGATAe*, is required for the terminal differentiation of the *Drosophila* endoderm.** *Dev Biol* 2005, **278:**576-586.
30. Zaffran S, Das G, Frasch M: **The NK-2 homeobox gene *scarecrow* (*scro*) is expressed in pharynx, ventral nerve cord and brain of *Drosophila* embryos.** *Mech Dev* 2000, **94:**237-241.
31. Goriely A, Stella M, Coffinier C, Kessler D, Mailhos C, Dessain S, Desplan C: **A functional homologue of *goosecoid* in *Drosophila*.** *Development* 1996, **122:**1641-1650.
32. Boyle MJ, Seaver EC: **Developmental expression of *foxA* and *gata* genes during gut formation in the polychaete annelid, *Capitella* sp. I.** *Evol Dev* 2008, **10:**89-105.
33. Arenas-Mena C, Wong KS: ***HeOtx* expression in an indirectly developing polychaete correlates with gastrulation by invagination.** *Dev Genes Evol* 2007, **217:**373-384.
34. Arenas-Mena C: **The transcription factors *HeBlimp* and *HeT-brain* of an indirectly developing polychaete suggest ancestral endodermal, gastrulation, and sensory cell-type specification roles.** *J Exp Zool Part B* 2008, **310:**567-576.
35. Arenas-Mena C: ***Brachyury*, *Tbx2/3* and *sall* expression during embryogenesis of the indirectly developing polychaete *Hydroides elegans*.** *Int J Dev Biol* 2013, **57:**73-83.
36. Arenas-Mena C: **Embryonic expression of *HeFoxA1* and *HeFoxA2* in an indirectly developing polychaete.** *Dev Genes Evol* 2006, **216:**727-736.
37. Arendt D, Technau U, Wittbrodt J: **Evolution of the bilaterian larval foregut.** *Nature* 2001, **409:**81-85.
38. Christodoulou F, Raible F, Tomer R, Simakov O, Trachana K, Klaus S, Snyman H, Hannon GJ, Bork P, Arendt D: **Ancient animal microRNAs and the evolution of tissue identity.** *Nature* 2010, **463:**1084-1088.
39. Tessmar-Raible K: **The evolution of sensory and neurosecretory cell types in bilaterian brains.** *Doctoral degree in Natural Sciences.* der Philipps-Universität Marburg, Vom Fachbereich Biologie; 2004.

40. Nederbragt AJ, te Welscher P, van den Driesche S, van Loon AE, Dictus WJ: **Novel and conserved roles for *orthodenticle/ otx* and *orthopedia/ otp* orthologs in the gastropod mollusc *Patella vulgata*.** *Dev Genes Evol* 2002, **212:**330-337.
41. Lartillot N, Lespinet O, Vervoort M, Adoutte A: **Expression pattern of *Brachyury* in the mollusc *Patella vulgata* suggests a conserved role in the establishment of the AP axis in Bilateria.** *Development* 2002, **129:**1411-1421.
42. Lartillot N, Le Gouar M, Adoutte A: **Expression patterns of *fork head* and *goosecoid* homologues in the mollusc *Patella vulgata* supports the ancestry of the anterior mesendoderm across Bilateria.** *Dev Genes Evol* 2002, **212:**551-561.
43. Dunn EF, Moy VN, Angerer LM, Angerer RC, Morris RL, Peterson KJ: **Molecular paleoecology: using gene regulatory analysis to address the origins of complex life cycles in the late Precambrian.** *Evol Dev* 2007, **9:**10-24.
44. Koinuma S, Umesono Y, Watanabe K, Agata K: **Planaria *FoxA* (*HNF3*) homologue is specifically expressed in the pharynx-forming cells.** *Gene* 2000, **259:**171-176.
45. Martin-Duran JM, Romero R: **Evolutionary implications of morphogenesis and molecular patterning of the blind gut in the planarian *Schmidtea polychroa*.** *Dev Biol* 2011, **352:**164-176.
46. Li X, Chuang C-K, Mao C-A, Angerer LM, Klein WH: **Two Otx Proteins Generated from Multiple Transcripts of a Single Gene in *Strongylocentrotus purpuratus*.** *Dev Biol* 1997, **187:**253-266.
47. Hinman VF, Nguyen AT, Davidson EH: **Expression and function of a starfish Otx ortholog, AmOtx: a conserved role for Otx proteins in endoderm development that predates divergence of the eleutherozoa.** *Mech Develop* 2003, **120:**1165-1176.
48. Livi CB, Davidson EH: **Expression and function of *blimp1/krox*, an alternatively transcribed regulatory gene of the sea urchin endomesoderm network.** *Dev Biol* 2006, **293:**513-525.
49. Shoguchi E, Satoh N, Maruyama YK: **Pattern of *Brachyury* gene expression in starfish embryos resembles that of hemichordate embryos but not of sea urchin embryos.** *Mech Dev* 1999, **82:**185-189.
50. Gross JM, McClay DR: **The role of Brachyury (T) during gastrulation movements in the sea urchin *Lytechinus variegatus*.** *Dev Biol* 2001, **239:**132-147.
51. Oliveri P, Walton KD, Davidson EH, McClay DR: **Repression of mesodermal fate by *foxa*, a key endoderm regulator of the sea urchin embryo.** *Development* 2006, **133:**4173-4181.
52. Hinman VF, Davidson EH: **Expression of a gene encoding a Gata transcription factor during embryogenesis of the starfish *Asterina miniata*.** *Gene Expr Patterns* 2003, **3:**419-422.
53. Lee PY, Davidson EH: **Expression of *Spgatae*, the *Strongylocentrotus purpuratus* ortholog of vertebrate GATA4/5/6 factors.** *Gene Expr Patterns* 2004, **5:**161-165.
54. Takacs CM, Amore G, Oliveri P, Poustka AJ, Wang D, Burke RD, Peterson KJ: **Expression of an NK2 homeodomain gene in the apical ectoderm defines a new territory in the early sea urchin embryo.** *Dev Biol* 2004, **269:**152-164.
55. Angerer LM, Oleksyn DW, Levine AM, Xiaotao L, Klein WH, Angerer RC: **Sea urchin *goosecoid* function links fate specification along the animal-vegetal and oral-aboral embryonic axes.** *Development* 2001, **128:**4393-4404.

56. Harada Y, Okai N, Taguchi S, Tagawa K, Humphreys T, Satoh N: **Developmental expression of the hemichordate *otx* ortholog.** *Mech Dev* 2000, **91:**337-339.
57. Tagawa K, Humphreys T, Satoh N: **Novel pattern of *Brachyury* gene expression in hemichordate embryos.** *Mech Dev* 1998, **75:**139-143.
58. Röttinger E, Martindale MQ: **Ventralization of an indirect developing hemichordate by NiCl(2) suggests a conserved mechanism of dorso-ventral (D/V) patterning in Ambulacraria (hemichordates and echinoderms).** *Dev Biol* 2011, **354:**173-190.
59. Taguchi S, Tagawa K, Humphreys T, Nishino A, Satoh N, Harada Y: **Characterization of a hemichordate *fork head*/*HNF-3* gene expression.** *Dev Genes Evol* 2000, **210:**11-17.
60. Takacs CM, Moy VN, Peterson KJ: **Testing putative hemichordate homologues of the chordate dorsal nervous system and endostyle: expression of *NK2.1* (*TTF-1*) in the acorn worm *Ptychodera flava* (Hemichordata, Ptychoderidae).** *Evol Dev* 2002, **4:**405-417.
61. Castro LF, Rasmussen SL, Holland PW, Holland ND, Holland LZ: **A Gbx homeobox gene in amphioxus: insights into ancestry of the ANTP class and evolution of the midbrain/hindbrain boundary.** *Dev Biol* 2006, **295:**40-51.
62. Onai T, Yu JK, Blitz IL, Cho KW, Holland LZ: **Opposing Nodal/Vg1 and BMP signals mediate axial patterning in embryos of the basal chordate amphioxus.** *Dev Biol* 2010, **344:**377-389.
63. Holland PW, Koschorz B, Holland LZ, Herrmann BG: **Conservation of *Brachyury* (*T*) genes in amphioxus and vertebrates: developmental and evolutionary implications.** *Development* 1995, **121:**4283-4291.
64. Shimeld SM: **Characterisation of amphioxus HNF-3 genes: conserved expression in the notochord and floor plate.** *Dev Biol* 1997, **183:**74-85.
65. Zhang Y-j, Mao B-y: **Developmental Expression of an Amphioxus (*Branchiostoma belcheri*) Gene Encoding a GATA Transcription Factor.** *Zool Res* 2009, **30:**137-143.
66. Venkatesh TV, Holland ND, Holland LZ, Su MT, Bodmer R: **Sequence and developmental expression of amphioxus *AmphiNk2-1*: insights into the evolutionary origin of the vertebrate thyroid gland and forebrain.** *Dev Genes Evol* 1999, **209:**254-259.
67. Hinman V, Degnan B: **Retinoic acid perturbs *Otx* gene expression in the ascidian pharynx.** *Dev Genes Evol* 2000, **210:**129-139.
68. Hudson C, Lemaire P: **Induction of anterior neural fates in the ascidian *Ciona intestinalis*.** *Mech Dev* 2001, **100:**189-203.
69. Satou Y, Imai KS, Satoh N: **Early embryonic expression of a LIM-homeobox gene *Cs-lhx3* is downstream of β-catenin and responsible for the endoderm differentiation in *Ciona savignyi* embryos.** *Development* 2001, **128:**3559-3570.
70. Bassham S, Postlethwait J: ***Brachyury* (*T*) expression in embryos of a larvacean urochordate, *Oikopleura dioica*, and the ancestral role of *T*.** *Dev Biol* 2000, **220:**322-332.
71. Corbo JC, Erives A, Di Gregorio A, Chang A, Levine M: **Dorsoventral patterning of the vertebrate neural tube is conserved in a protochordate.** *Development* 1997, **124:**2335-2344.

72. Imai KS, Hino K, Yagi K, Satoh N, Satou Y: **Gene expression profiles of transcription factors and signaling molecules in the ascidian embryo: towards a comprehensive understanding of gene networks.** *Development* 2004, **131:**4047-4058.
73. Ragkousi K, Beh J, Sweeney S, Starobinska E, Davidson B: **A single GATA factor plays discrete, lineage specific roles in ascidian heart development.** *Dev Biol* 2011, **352:**154-163.
74. Ristoratore F, Spagnuolo A, Aniello F, Branno M, Fabbrini F, Di Lauro R: **Expression and functional analysis of *Cititf1*, an ascidian *NK-2* class gene, suggest its role in endoderm development.** *Development* 1999, **126:**5149-5159.
